# TORC1 regulates the transcriptional response to glucose and developmental cycle via the Tap42-Sit4-Rrd1/2 pathway in *Saccharomyces cerevisiae*

**DOI:** 10.1186/s12915-021-01030-3

**Published:** 2021-05-06

**Authors:** Mohammad Alfatah, Jin Huei Wong, Vidhya Gomathi Krishnan, Yong Cheow Lee, Quan Feng Sin, Corinna Jie Hui Goh, Kiat Whye Kong, Wei Ting Lee, Jacqueline Lewis, Shawn Hoon, Prakash Arumugam

**Affiliations:** 1 Bioinformatics Institute, 30 Biopolis Street, Singapore, 138671 Singapore; 2 Molecular Engineering Lab, 61 Biopolis Drive, Singapore, 138673 Singapore; 3 Singapore Institute for Food and Biotechnology Innovation, 31 Biopolis Way, #01-02 Nanos, Singapore, 138669 Singapore; 4 School of Biological Sciences, Nanyang Technological University, 60 Nanyang Drive, Singapore, 637551 Singapore

**Keywords:** Transcriptional response to glucose, TORC1, Tap42/Sit4/Rrd1-2 module, Spore germination

## Abstract

**Background:**

Target of Rapamycin Complex 1 (TORC1) is a highly conserved eukaryotic protein complex that couples the presence of growth factors and nutrients in the environment with cellular proliferation. TORC1 is primarily implicated in linking amino acid levels with cellular growth in yeast and mammals. Although glucose deprivation has been shown to cause TORC1 inactivation in yeast, the precise role of TORC1 in glucose signaling and the underlying mechanisms remain unclear.

**Results:**

We demonstrate that the presence of glucose in the growth medium is both necessary and sufficient for TORC1 activation. TORC1 activity increases upon addition of glucose to yeast cells growing in a non-fermentable carbon source. Conversely, shifting yeast cells from glucose to a non-fermentable carbon source reduces TORC1 activity. Analysis of transcriptomic data revealed that glucose and TORC1 co-regulate about 27% (1668/6004) of yeast genes. We demonstrate that TORC1 orchestrates the expression of glucose-responsive genes mainly via the Tap42-Sit4-Rrd1/2 pathway. To confirm TORC1’s function in glucose signaling, we tested its role in spore germination, a glucose-dependent developmental state transition in yeast. TORC1 regulates the glucose-responsive genes during spore germination and inhibition of TORC1 blocks spore germination.

**Conclusions:**

Our studies indicate that a regulatory loop that involves activation of TORC1 by glucose and regulation of glucose-responsive genes by TORC1, mediates nutritional control of growth and development in yeast.

**Supplementary Information:**

The online version contains supplementary material available at 10.1186/s12915-021-01030-3.

## Background

Cells sense changes in nutrient availability in their environment and accordingly adjust their growth and developmental cycles. Glucose response and spore germination in *Saccharomyces cerevisiae* are excellent model systems to study this biological phenomenon. When glucose is added to yeast cells growing in a non-fermentable carbon source, the transcriptome and metabolome are extensively reprogrammed to facilitate their growth in the new milieu. This adaptation is referred to as the “transcriptional response to glucose” (will be referred to as glucose response here). Likewise, when haploid spores are transferred to a nutrient medium that contains glucose (or a rapidly fermentable carbon source), they exit their state of ‘hibernation’ and re-enter the mitotic cell cycle [1]. Exactly how changes in glucose levels in the environment are sensed by the cell leading to dramatic modulation of growth and developmental regulatory circuits is poorly understood.

**Fig. 1:**
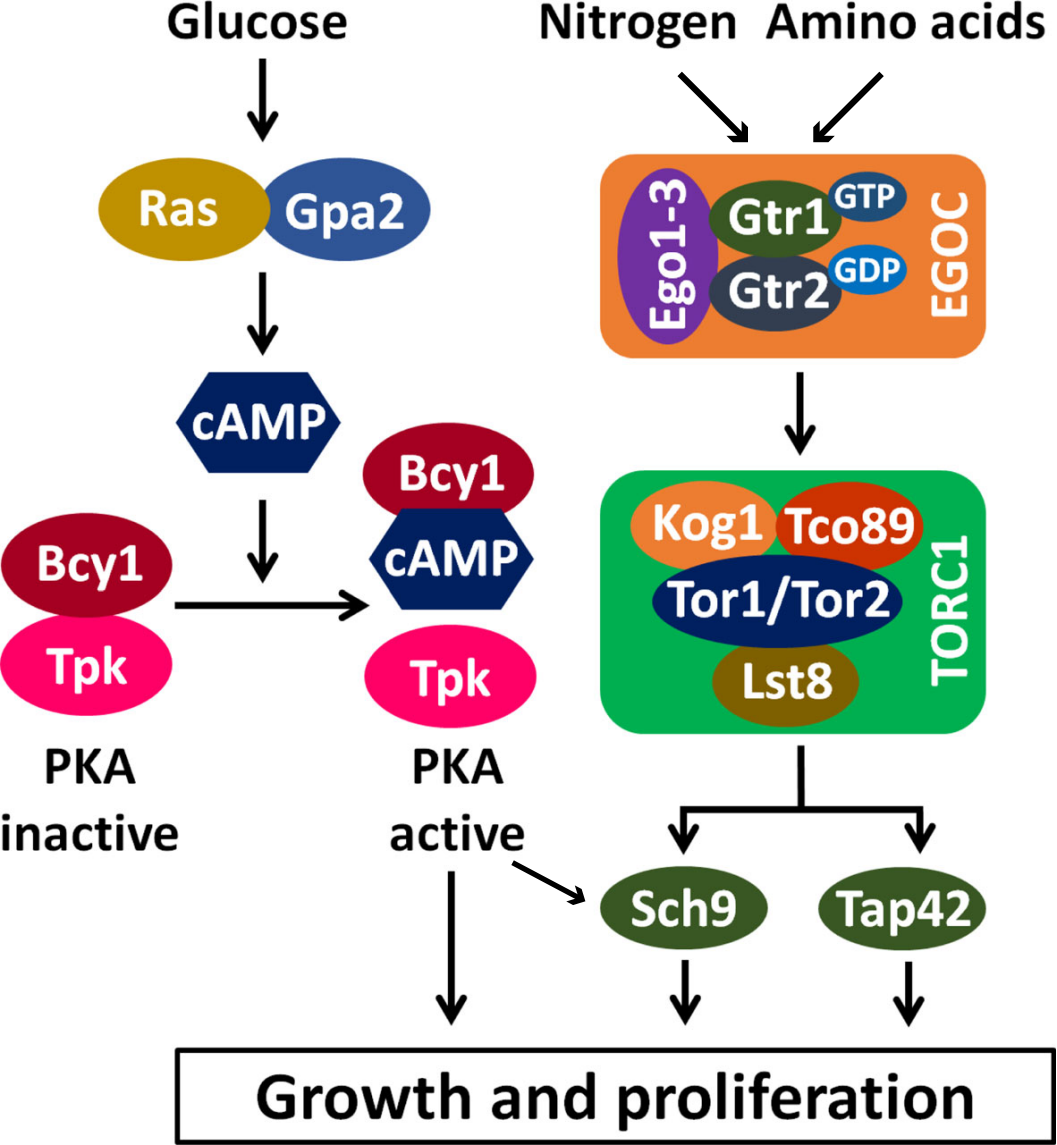
PKA and TORC1 connect the presence of glucose and amino acids/nitrogen levels respectively with cell proliferation in yeast. See the introduction for details

In yeast, glucose levels in the environment are thought to be mainly sensed by the cyclic AMP (cAMP)-dependent protein kinase A (PKA) [2]. Addition of glucose or a rapidly fermentable sugar to the medium activates PKA via GTP-binding proteins Ras1/2 and Gpa2 which results in activation of adenylate cyclase and cAMP production (Fig. 1). PKA consists of a catalytic subunit (Tpk1, 2, or 3) and a regulatory subunit Bcy1. Bcy1 inhibits PKA’s catalytic activity, and this inhibition is relieved by binding of cAMP to Bcy1 causing its dissociation from the catalytic subunit (Fig. 1). PKA phosphorylates several cellular proteins leading to enhanced protein synthesis and inhibition of stress response.

While glucose signaling is mainly attributed to PKA, the coupling of amino acid levels and quality of nitrogen source with cellular growth is performed by TORC1, a multi-subunit protein complex conserved among eukaryotes [3]. TORC1 promotes anabolic processes such as protein synthesis and inhibits catabolic processes like autophagy. TORC1 in *Saccharomyces cerevisiae* is composed of four subunits, namely Tor1/Tor2 (a serine-threonine kinase), Lst8 (equivalent of mLst8/GβL), Kog1 (equivalent of mammalian raptor), and Tco89 (a yeast-specific subunit). The conserved Rag GTPases Gtr1 and Gtr2 act upstream of TORC1 and link the amino acid and nitrogen levels with TORC1 activity (Fig. 1). Gtr1 and Gtr2 form a heterodimer and the state of the nucleotides bound to Gtr1-Gtr2 determines whether they activate or inhibit TORC1. Specifically, a heterodimer of Gtr1/Gtr2 in which the Gtr1 is bound to GTP and Gtr2 is bound to GDP, activates TORC1. Binding of Gtr1/Gtr2 to TORC1 is facilitated by three proteins Ego1/Meh1, Ego2, and Ego3/Slm4, which together with Gtr1/Gtr2 constitute the EGO complex. TORC1 links changes in nutrient levels with transcriptomic reprogramming via its downstream effectors, namely Sch9 (a serine-threonine kinase) and Tap42 (a PP2A phosphatase-binding protein).

During the glucose response, yeast cells reshape their metabolism by switching from oxidative phosphorylation to glycolysis to obtain energy. About 40% of yeast genes are altered in their expression upon glucose addition and 90% of these transcriptional changes could be induced by activation of either Ras2 or Gpa2 [4]. Furthermore, an activated form of PKA recapitulated 90% of transcriptomic changes induced by glucose indicating that PKA is the main regulator of the transcriptional response to glucose in yeast [5].

TORC1’s role in regulating the glucose response is not clear. A transcriptomic study showed that PKA works along with TORC1 in regulating the transcriptional response to glucose [6]. However, another study found that the major TORC1 effector Sch9 plays a very minor role in the glucose response [5]. Although activation of TORC1-effector kinase Sch9 recapitulated a number of transcriptional changes induced by glucose, inactivating Sch9 had only a minor effect on the glucose response [5]. It is also unclear whether the role of Sch9 in glucose response is downstream of TORC1 activation or independent of TORC1 activity. Thus, it is very important to clarify TORC1’s precise function in the glucose response.

In this paper, we show that TORC1 activity is upregulated during the glucose response. TORC1 is activated by glucose through Gtr1/Gtr2-dependent and Gtr1/Gtr2-independent mechanisms. Transcriptomic analysis revealed that about 50% of glucose-responsive genes are regulated by TORC1. We show that TORC1 activity is required for establishment and maintenance of transcriptional response to glucose. TORC1 regulates the glucose-responsive genes through its downstream effectors Tap42/Sit4/Rrd1/2. Our results are consistent with the model that inhibition of TORC1 leads to activation of Sit4/Rrd1-Rrd2 phosphatase which effects changes in the expression of glucose-responsive genes. If TORC1’s role in glucose response is physiologically relevant, then TORC1 inhibition should affect spore germination, a glucose-dependent developmental state transition in yeast [1]. This prediction was confirmed by our observations that TORC1 regulates the glucose-responsive genes during spore germination and is essential for spore germination. We propose that TORC1 is an important regulator of the glucose response and this function is essential for developmental state transition from quiescent spores into actively growing vegetative cells.

## Results

### Glucose is necessary and sufficient for TORC1 activation

**Fig. 2:**
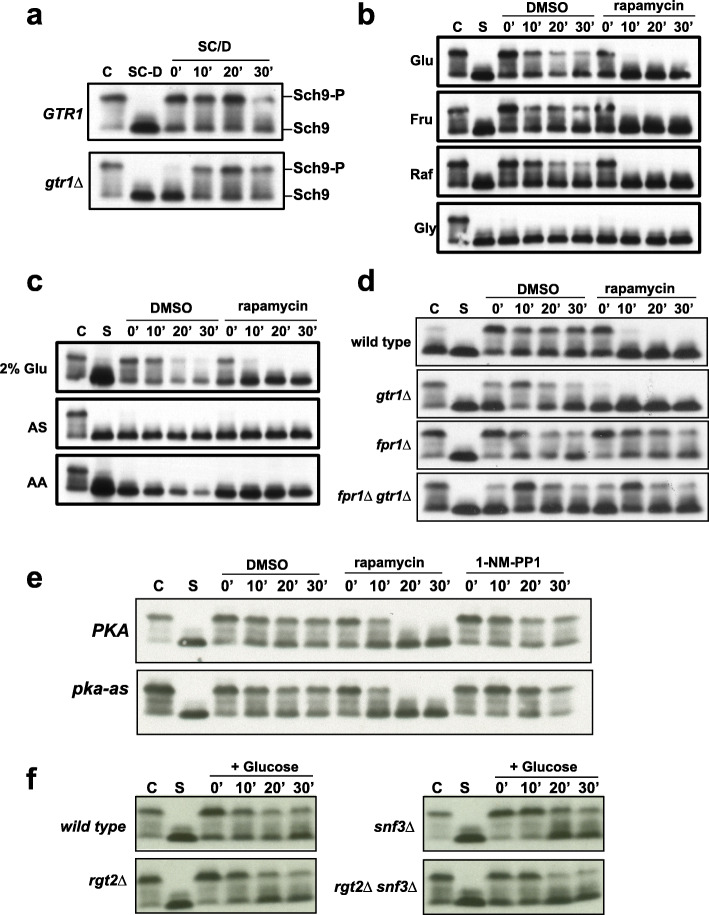
Presence of glucose in the medium is necessary and sufficient for TORC1 activation. **a** Log-phase wild type and *gtr1Δ* cells (C) grown in synthetic medium with 2% glucose (SC/D) were transferred into synthetic medium lacking glucose (SC-D) and incubated for 1 h. Glucose-starved cells (SC-D) were then transferred back into SC medium (SC/D). Aliquots of the yeast cultures were taken after 0’, 10’, 20’, and 30’ and used for preparing protein extracts. Phosphorylation of Sch9 was monitored by Western blotting. **b** Wild type cells in logarithmic phase (C) were subjected to complete nutrient starvation by incubating them in 0.3 M sorbitol for 1 h. Starved cells (S) were then transferred to a solution containing either 110 mM glucose or 110 mM fructose, or 110 mM raffinose or 110 mM glycerol in the presence and absence of rapamycin (2 μM). Aliquots of the cultures were taken after 0’, 10’, 20’, and 30’ and used for preparing protein extracts. Phosphorylation of Sch9 was monitored by Western blotting. **c** Wild type cells in logarithmic phase (C) were subjected to complete nutrient starvation by incubating them in 0.3 M sorbitol for 1 h. Starved cells (S) were then transferred to a solution containing either 110 mM glucose or ammonium sulfate or amino acid mixture in the presence and absence of rapamycin (2 μM). Aliquots of the cultures were taken after 0’, 10’, 20’, and 30’ and used for preparing protein extracts. Phosphorylation of Sch9 was monitored by Western blotting. **d** Wild type, *gtr1Δ*, *fpr1Δ*, and *fpr1Δ gtr1Δ* cells in log phase were subjected to complete nutrient starvation by incubating them in 0.3 M sorbitol for 1 h. They were then transferred to a 2% glucose solution in the presence and absence of rapamycin (2 μM). Aliquots of the cultures were taken after 0’, 10’, 20’, and 30’ and used for preparing protein extracts. Phosphorylation of Sch9 was monitored by Western blotting. **e** Wild type and *pka-as* cells subjected to complete nutrient starvation were transferred to 2% glucose solution in the presence of either DMSO or rapamycin (2 μM) or 1-NM-PP1 (1.5 μM). Aliquots of the cultures were taken after 0’, 10’, 20’, and 30’ and used for preparing protein extracts. Phosphorylation of Sch9 was monitored by Western blotting. **f** Wild type, *rgt2Δ*, *snf3 Δ*, and *rgt2Δ snf3Δ* cells subjected to complete starvation were transferred to 2% glucose solution. Aliquots of the cultures were taken after 0’, 10’, 20’, and 30’ and used for preparing protein extracts. Phosphorylation of Sch9 was monitored by Western blotting

To investigate the role of TORC1 in glucose signaling, we first tested whether glucose is required for TORC1 activation. To assess the kinetics of TORC1 activation, we tagged the TORC1 substrate Sch9 with 6 copies of the hemagglutinin (HA) epitope. Phosphorylation of Sch9 can be assayed by cleaving it with NTCB (2-nitro-5-thiocyanatobenzoic acid) followed by detecting the electrophoretic mobility of the C-terminal HA-tagged Sch9 fragment by Western analysis [7]. We transferred log-phase wild type and *gtr1*Δ cells grown in Synthetic Complete medium containing 2% glucose (SC/D) into SC medium lacking glucose (SC-D). After 60’ following transfer, Sch9 was completely dephosphorylated in both wild type and *gtr1*Δ cells indicating that TORC1 activity requires the presence of glucose in the medium (Fig. 2a). We transferred the glucose-starved cells back into SC medium-containing glucose (SC/D). TORC1 was reactivated immediately upon addition of glucose in the wild type strain (Fig. 2a). However, TORC1 activation in the *gtr1*Δ strain was delayed by about 10 minutes in comparison to the wild type strain (Fig. 2a). These results suggest that glucose activates TORC1 via Gtr1/2-dependent and Gtr1/2-independent pathways in yeast.

We then tested whether glucose is sufficient for TORC1 activation. We subjected log-phase yeast cells to complete nutrient starvation by washing off all the nutrients and transferring them into 0.3 M sorbitol. TORC1 was inactive after 1 h of incubation in 0.3 M sorbitol (Fig. 2b). We then added either glucose (110 mM) or equimolar amounts of fructose or raffinose or glycerol to starved cells, in the presence and absence of TORC1 inhibitor rapamycin (2 μM). Addition of glucose or fructose or raffinose caused an immediate phosphorylation of Sch9 which was abolished by addition of rapamycin (2 μM) (Fig. 2b). However, addition of glycerol did not activate TORC1. Unlike glucose, both ammonium sulfate and a mixture of all amino acids failed to activate TORC1 in the assay (Fig. 2c). Our results indicate that glucose (or a rapidly fermenting carbon source like fructose/raffinose) is sufficient for TORC1 activation.

We then tested whether glucose-induced TORC1 activation requires its upstream regulator Gtr1. Addition of glucose caused phosphorylation of Sch9 immediately in wild type cells but with a 10-min delay in *gtr1Δ* cells (Fig. 2d). Sch9 phosphorylation was abolished by rapamycin (2 μM) in both wild type and *gtr1Δ* cells (Fig. 2d). Rapamycin binds to the peptidyl-prolyl cis-trans isomerase Fpr1, and Rapamycin-Fpr1 complex inhibits TORC1 by binding to Tor1/Tor2 kinase [8]. To determine whether rapamycin at 2 μM specifically inhibits TORC1, we examined the effect of rapamycin on glucose-induced Sch9 phosphorylation in wild type and *gtr1Δ* strains lacking *FPR1* gene*.* As observed previously, there was a 10-min delay in the onset of Sch9 phosphorylation in the *fpr1Δ gtr1*Δ strain in comparison to the *fpr1Δ* strain. However, addition of rapamycin had no effect on Sch9 phosphorylation in both strains confirming that TORC1 is the Sch9-phosphorylating kinase. In summary, our data indicate that glucose is sufficient to activate TORC1 through Gtr1-dependent and Gtr1-independent mechanisms in yeast.

### Glucose-induced TORC1 activation is independent of PKA and glucose sensors Snf2 and Rgt2

As PKA is the main regulator of the transcriptional response to glucose in yeast [2], we tested whether glucose-induced TORC1 activation requires PKA activity. Catalytic subunit of PKA is encoded by three genes *TPK1-3* in yeast. We constructed an analog-sensitive allele of PKA (*pka-as*) by deleting *TPK3* and introducing gatekeeper mutations namely *tpk1-*M164G and *tpk2-*M147G [5]. 1-NM-PP1 completely inhibited the growth of the *pka-as* strain at 1.5 μM but did not affect the wild type strain indicating that it specifically inhibits PKA activity in the *pka-as* strain (Additional file 1: Fig. S1a). This was confirmed by monitoring the effect of 1-NM-PP1 on phosphorylation of PKA substrates using an anti-PKA substrate antibody. Addition of 1-NM-PP1 (Concentration range 1.5–25 μM) to *pka-as* but not wild type (*PKA*) cells inhibited PKA activity (Additional file 1: Fig. S1b). We tested whether PKA activity is required for glucose-induced TORC1 activation by adding 1-NM-PP1 to wild type and *pka-as* cells during complete nutrient starvation and following addition of 2% glucose. As expected, 1-NM-PP1 at 1.5 μM inhibited PKA activity in *pka-as* but not *PKA* cells (Additional file 1: Fig. S2). Rapamycin (2 μM) had no discernible effect on PKA activity (Additional file 1: Fig. S2). Glucose-induced Sch9 phosphorylation in 1-NM-PP1-treated *PKA* and *pka-as* cells were comparable but was completely inhibited by rapamycin (Fig. 2e). These results indicate that glucose-induced TORC1 activation is independent of PKA activity. We also tested whether glucose-induced TORC1 activation is dependent on glucose sensors namely Rgt2 and Snf3 that regulate the transport of glucose into yeast cells [2]. Glucose-induced TORC1 activation was comparable in wild type, *rgt2Δ*, *snf3Δ*, and *rgt2Δ snf3Δ* strains (Fig. 2f) precluding a role for these glucose sensors in glucose-induced TORC1 activation.

### TORC1 is activated during glucose response

**Fig. 3:**
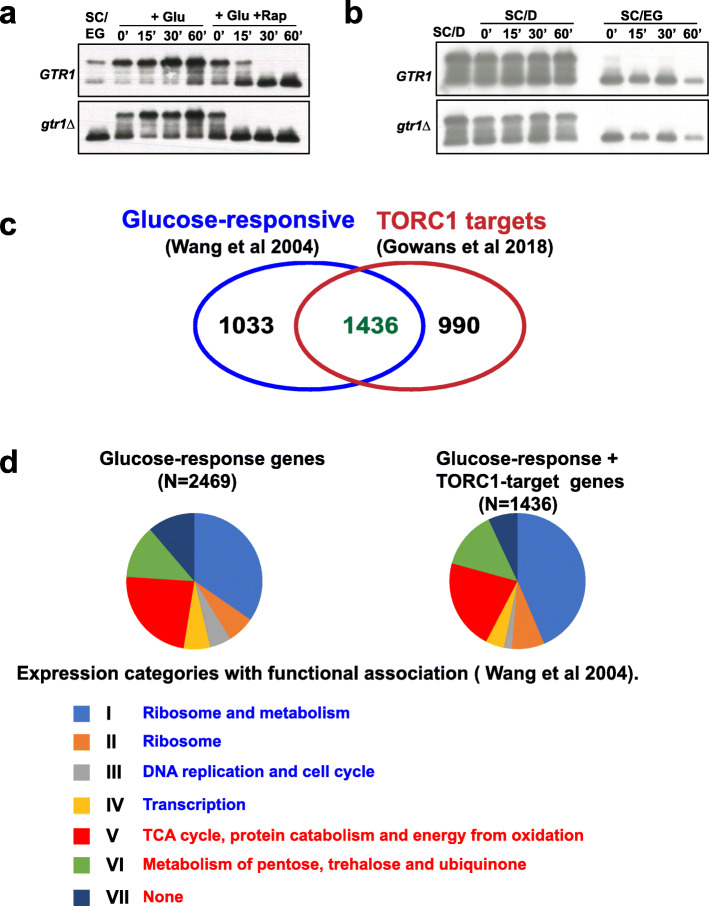
TORC1 activity increases during glucose response. **a** Wild type and *gtr1Δ* cells were grown to logarithmic phase in SC/EG medium, and then glucose (2% final concentration) was added to the cultures in the presence of either DMSO or rapamycin (200 nM). Aliquots of the cultures were taken after 0’, 15’, 30’, and 60’ and used for preparing protein extracts. Phosphorylation of Sch9 was monitored by Western blotting. **b** Wild type and *gtr1Δ* cells were grown to logarithmic phase in SC/D medium. Cultures were then divided into two parts. For one part, cells were pelleted and washed thrice with SC/EG medium, resuspended in SC/EG medium, and incubated at 30 °C. The second part was transferred back to SC/D and was also incubated at 30 °C. Aliquots of the cultures were taken after 0’, 15’, 30’, and 60’ and used for preparing protein extracts. Phosphorylation of Sch9 was monitored by Western blotting. **c** Venn diagram showing the overlap of glucose-responsive genes [4] with TORC1 target genes [9]. **d** Pie chart shows the distribution of the glucose-responsive genes and TORC1-glucose co-regulated (TGC) genes among the various gene clusters defined by response to glucose and Ras activation [4]. Functional enrichment among the various clusters induced and repressed by glucose are indicated, in blue and red fonts respectively

When glucose is added to yeast cells growing in a medium containing a non-fermentable carbon source, several genes are induced or repressed which restructure the transcriptional and metabolic state of yeast [4]. This phenomenon is referred to as the glucose response and the differentially expressed genes constitute the glucose-responsive genes. We investigated whether TORC1 activity is altered during the glucose response. We grew wild type and *gtr1Δ* cells to log phase in SC medium containing non-fermentable carbon sources ethanol and glycerol (SC/EG) and added glucose to a final concentration of 2% along with either DMSO or rapamycin (200 nM). Sch9 phosphorylation increased upon addition of glucose to wild type and *gtr1Δ* cells growing in in SC/EG medium and abolished by rapamycin treatment after 30’ (Fig. 3a). Increase in TORC1 activity upon glucose addition was delayed in *gtr1Δ* cells as observed previously in our glucose-induced TORC1 activity assays (Fig. 2a, d). We also performed the converse experiment in which we switched the carbon source of log-phase yeast cells from glucose to ethanol-glycerol. TORC1 activity was drastically reduced upon transfer from SC/D to SC/EG in both wild type and *gtr1*Δ cells (Fig. 3b).

### Overlap of TORC1 targets with the glucose-responsive genes

We then explored whether there is any overlap of glucose-responsive genes with TORC1 target genes by analyzing published transcriptomic data. Based on the response to glucose and Ras activation, glucose-responsive genes (*N* = 3273) were classified into 8 categories I–VIII [4]. Genes belonging to categories I–IV and categories V–VII are activated and repressed by glucose, respectively [4]. Genes in class VIII (*N* = 804) are not regulated by glucose in wild type cells but only in *pka-as* cells and were therefore excluded from our analysis. We compared the remaining list of glucose-responsive genes (*N* = 2469) with the list of 2426 TORC1 target genes reported in a recent transcriptomic study [4, 9]. We found that 58% of glucose-responsive genes (*N* = 1436; upregulated = 828 and downregulated = 608) were co-regulated by TORC1 (Fig. 3c). Among the 828 genes upregulated by glucose, 807 (98%) genes were positively regulated by TORC1. Likewise, of the 608 genes negatively regulated by glucose, 578 (95%) genes were also negatively regulated by TORC1. Genes regulated by TORC1 were spread across the seven categories of glucose-responsive genes to different extents (Fig. 3d). These observations indicate that addition of glucose and TORC1 activation have similar qualitative effects on the yeast transcriptome.

### TORC1 is required for establishment and maintenance of TGC gene expression

We investigated whether TORC1 is required for the transcriptional response to glucose.

**Fig. 4:**
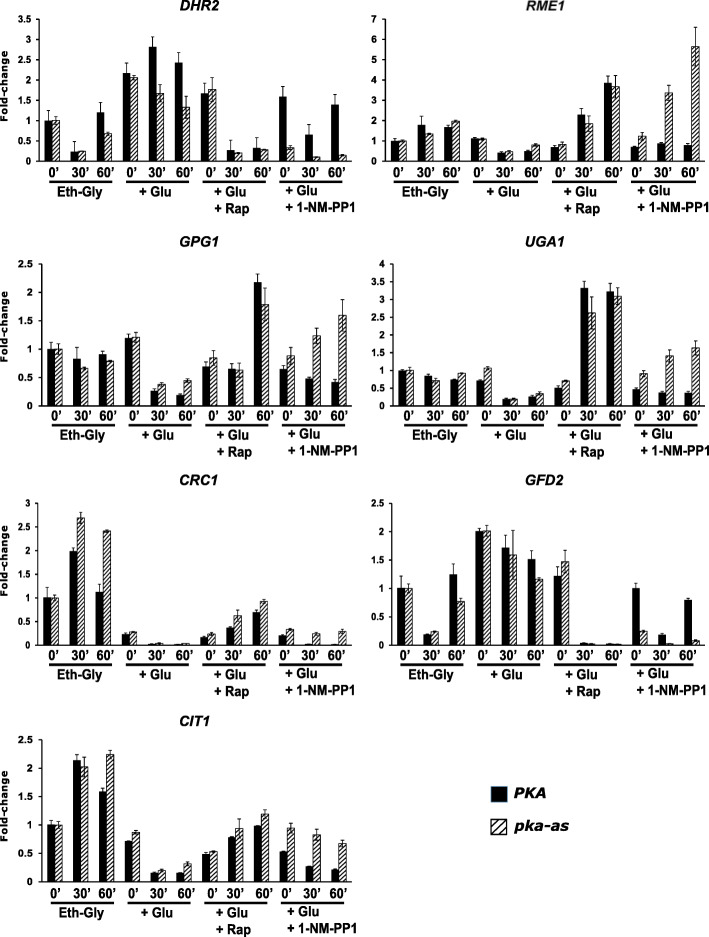
TORC1 and PKA co-regulate the transcriptional response to glucose. Wild type (PKA) and *pka-as* cells were grown to logarithmic phase were grown to logarithmic phase in SC/EG medium and then glucose (2% final concentration) was added in the presence of either rapamycin (200 nM) or 1-NM-PP1 (1.5 μM) or DMSO. Aliquots of the cultures were taken after 0, 30’, and 60’. RNA was extracted from the cultures, and the expression of the indicated 7 TGC genes were analyzed by real-time qRT-PCR. Data are presented as means ± standard deviation (*n* = 2 technical replicates). Data from two additional biological replicates of this experiment are presented in Fig. S3

We chose 7 TORC1 target genes *GFD2* (I), *DHR2* (II), *CIT1*(V), *CRC1*(V), *UGA1*(VI), *RME1*(VI), and *GPG1*(VII) spread across the top 5 expression categories as representative of “TORC1 and Glucose Co-regulated (TGC)” genes for analysis. The choice of these 7 genes was also informed by transcriptome analysis of TORC1 targets during spore germination (see below). We tested whether TORC1 activity is necessary for glucose-induced changes in expression of the seven TGC genes. For comparison, we tested the role of PKA activity in the transcriptional response to glucose using the *pka-as* strain. We added glucose (to a final concentration of 2%) to log-phase wild type (*PKA*) and *pka-as* cells growing in SC/EG (SC medium containing 2% ethanol and 2% glycerol), in the presence of either DMSO or rapamycin (200 nM) or 1-NM-PP1 (1.5 μM). Expression of all the 7 TGC genes was significantly altered after 30 min following addition of glucose (Fig. 4 and Additional file 1: Fig. S3). As expected, *GFD2* and *DHR2* genes (belonging to clusters I and II respectively) were upregulated in the presence of glucose (Fig. 4, Additional file 1: Fig. S3 and Additional file 2: Table S1) in both *PKA* and *pka-as* cells. Genes *GPG1*, *UGA1*, *RME1*, *CIT1*, and *CRC1* (belonging to clusters V–VII*)* were downregulated in the presence of glucose (Fig. 4, Additional file 1: Fig. S3 and Additional file 2: Table S1). Glucose-induced changes in the TGC genes were inhibited by 1-NM-PP1 in *pka-as* cells but not in wild type cells (Fig. 4, Additional file 1: Fig. S3, and Additional file 2: Table S1). There was a non-specific effect of 1-NM-PP1 on expression of *DHR2* and *GFD2* (Fig. 4 and Additional file 1: Fig. S3). However, 1-NM-PP1 still affected *DHR2* and *GFD2* expression much more strongly in *pka-as* cells compared to wild type cells (Fig. 4 and Additional file 1: Fig. S3). These results confirm PKA’s role in the transcriptional response to glucose. Importantly, glucose-induced changes in expression of the 7 TGC genes were also inhibited by addition of rapamycin (Fig. 4, Additional file 1: Fig. S3 and Additional file 2: Table S1). Timing of change in transcript levels in the presence of rapamycin (30’) agrees with timing of TORC1 inhibition observed previously (Fig. 3a). These results indicate that TORC1 and PKA co-regulate the transcriptional response to glucose in yeast.

We then tested whether maintenance of TGC gene expression in glucose-containing medium is dependent on TORC1 activity. We treated log-phase yeast cells growing in YPD medium (with 2 % glucose) with either DMSO or rapamycin (200 nM) and analyzed the expression of the 7 TGC genes by real-time qRT-PCR. Addition of rapamycin affected the expression of all the 7 genes with the transcript levels shifting towards the corresponding expression level in SC/EG medium (Additional file 3: Fig. S4). These results indicate that TORC1 activity is also required for maintaining the expression status of TGC genes during growth in glucose-containing medium (Additional file 3: Fig. S4).

### TORC1 regulates the expression of glucose-responsive genes independently of Bcy1 phosphorylation

TORC1 could regulate the expression of glucose-responsive genes indirectly by activating PKA. Indeed, TORC1 has been shown to promote PKA activity by inhibiting phosphorylation of PKA inhibitor Bcy1 at T129 [10]. Phospho-mimetic mutation of T129 (*bcy1-T129D*) was shown to have an inhibitory effect on PKA activity [10]. If TORC1’s effect on PKA activity via regulating T129 phosphorylation is important for the transcriptional response to glucose, then *bcy1-T129D* should block TORC1’s role in the glucose response. We added glucose (final concentration = 2%) to *BCY1* and *bcy1-T129D* cultures growing in SC/EG medium and monitored the expression of three TGC genes *DHR2*, *CIT1*, and *RME1* by real-time qRT-PCR. Rapamycin treatment affected the expression of 3 TGC genes to comparable extents in both wild type and *bcy1-T129D* strains (Additional file 3: Fig. S5) indicating that TORC1 regulates the glucose-responsive genes independently of Bcy1 phosphorylation at T129.

### TORC1 inhibition does not affect PKA activity

TORC1 and PKA have been shown to exert mutually antagonistic effects on their activities [11]. Our previous data indicated TORC1 inhibition had no effect on PKA activity in yeast cells treated with 2% glucose following complete starvation (Fig. 2e and Additional file 1: Fig. S2). To check if this result also extends to actively growing cells, we grew wild type and *pka-as* cells to logarithmic phase and treated them with either DMSO or rapamycin or 1-NM-PP1. We assayed PKA activity by Western blotting of whole cell extracts using a phospho-specific antibody directed against phosphorylated PKA substrates [10]. As expected, 1-NM-PP1 inhibited phosphorylation of PKA substrates in *pka-as* cells but not in wild type cells (Additional file 3: Fig. S6a). In contrast, rapamycin treatment did not affect phosphorylation of PKA substrates (Additional file 3: Fig. S6a). However, rapamycin treatment activated expression of TORC1-repressed genes *DIP5* and *GAP1* confirming inhibition of TORC1 (Additional file 3: Fig. S6b). Our data suggest that TORC1 inhibition does not affect PKA activity.

### TORC1 regulates expression of glucose-responsive genes independently of Sch9

**Fig. 5:**
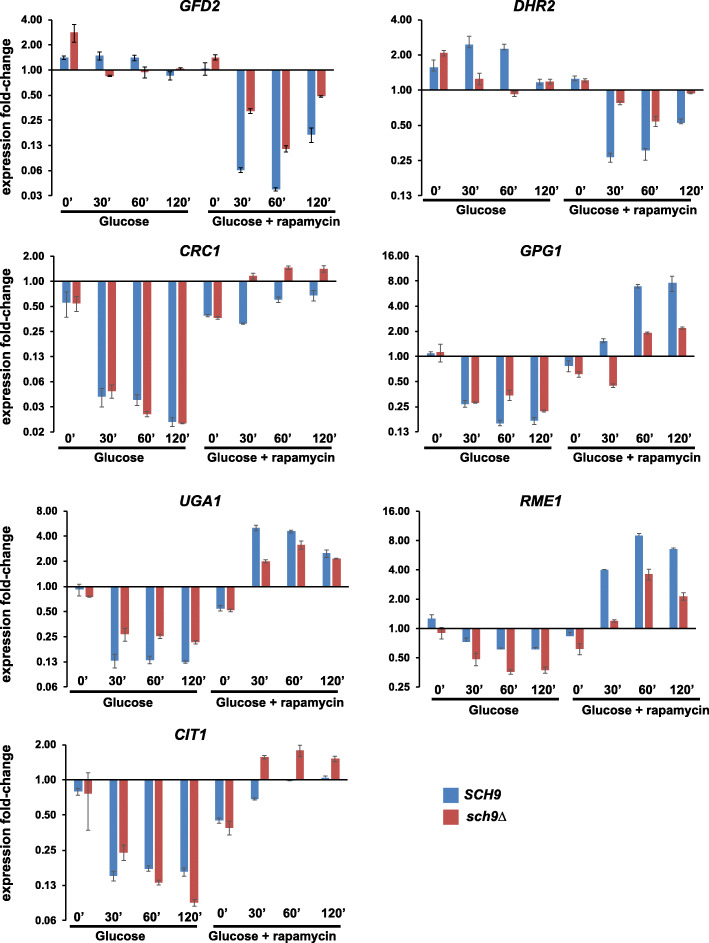
TORC1 regulates the expression of glucose-responsive genes independently of Sch9. Wild type and *sch9Δ* cells were grown to logarithmic phase in SC/EG medium and then glucose (2% final concentration) was added to the cultures in the presence of either rapamycin (200 nM) or DMSO. Aliquots of the cultures were taken after 0’, 30’, 60’, and 120’. RNA was extracted from the cultures and the expression of the indicated 7 TGC genes (*GFD2*, *GPG1*, *UGA1*, *RME1*, *CIT1*, *CRC1*, and *DHR2*) were analyzed by real-time qRT-PCR. Data are presented as means ± standard deviation (*n* = 2 technical replicates). Data from two additional biological replicates of this experiment are presented in Fig. S7

As TORC1 appears to regulate the transcriptional response to glucose independently of PKA, we focused our attention on its two downstream effectors Sch9 and Tap42. As PKA and TORC1 co-regulate the glucose response, inactivating the TORC1 effector involved in glucose response is not expected to have a major effect on glucose-induced gene expression. However, rapamycin treatment will have a reduced effect on expression of glucose-responsive genes in the effector mutant strain in comparison to the wild type strain. To assess the role of Sch9 in TORC1-mediated regulation of the TGC genes, we added glucose (2% final) to wild type and *sch9Δ* cultures growing in SC/EG medium. We assayed the expression of the 7 TGC genes in the presence and absence of rapamycin by real-time qRT-PCR. The 7 TGC genes were either upregulated (*GFD2* and *DHR2*) or downregulated (*CIT1*, *CRC1*, *UGA1*, *RME1*, and *GPG1*) in both wild type and *sch9Δ* strains following the addition of glucose to the medium (Fig. 5, Additional file 2: Table S1 and Additional file 3: Fig. S7). Importantly, addition of rapamycin reversed the glucose-induced changes in TGC gene expression albeit to different extents in wild type and *sch9Δ* strains. Doubling time of *sch9Δ* cells is twice that of wild type cells (Additional file 3: Fig. S8). Quantitative differences in the effect of rapamycin on TGC gene expression could be caused by pleiotropic growth defects of *sch9Δ*. Taken together, our data indicate that TORC1 regulates the glucose-responsive genes independently of Sch9. However, we cannot exclude the possibility that Sch9 facilitates the regulation of glucose-responsive genes either via TORC1 or independently of TORC1.

### TORC1 regulates expression of glucose-responsive genes via Tap42

**Fig. 6:**
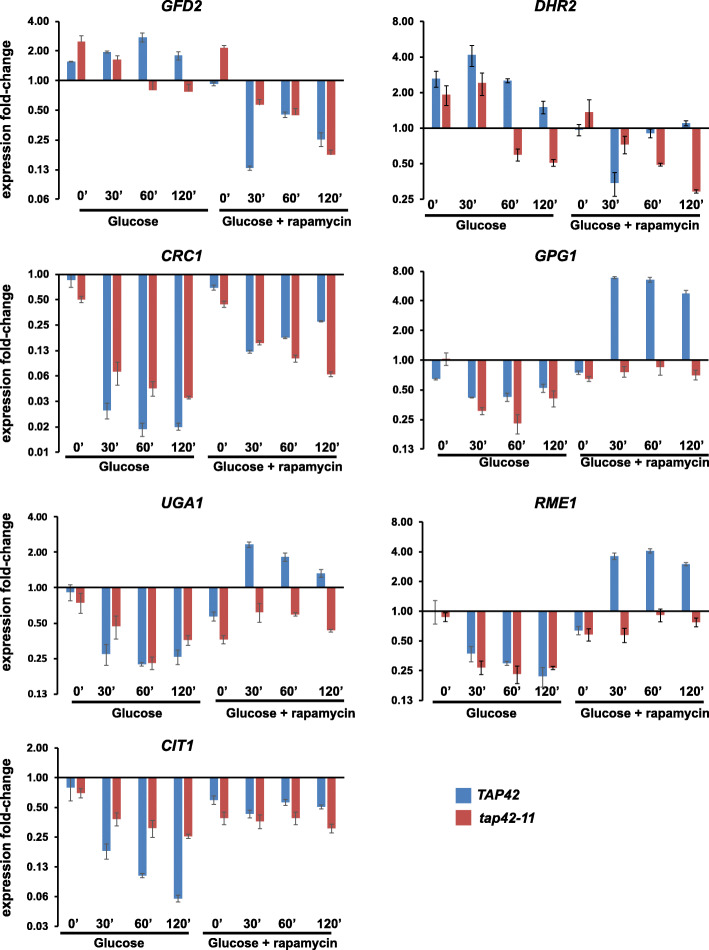
Regulation of glucose-responsive genes by TORC1 is dependent on Tap42. Wild type or *tap42-11* cells were grown to logarithmic phase at 25 °C (permissive temperature) in SC/EG medium and then shifted to 37 °C for 30’ to inactivate *tap42-11*. Glucose (2% final concentration) was added to the cultures in the presence of either rapamycin (200 nM) or DMSO. Aliquots of the cultures were taken after 0’, 30’, 60’, and 120’. RNA was extracted from the cultures and the expression of the 7 TGC genes (*GFD2*, *GPG1*, *UGA1*, *RME1*, *CIT1*, *CRC1*, and *DHR2*) were analyzed by real-time qRT-PCR. Data are presented as means ± standard deviation (*n* = 2 technical replicates). Data from two additional biological replicates of this experiment are presented in Fig. S9

To test the role of Tap42 in the expression of glucose-responsive genes, we used *tap42-11* a temperature-sensitive allele of *TAP42* [12]. We grew wild type and *tap42-11* cells to mid-log phase at 25 °C (permissive temperature) in SC/EG medium and transferred them to 37 °C (non-permissive temperature) for 30’ to inactivate Tap42. We then added 2% glucose to the cultures along with either rapamycin or DMSO and assayed the expression of the 7 TGC genes by real-time qRT-PCR. Induction of *GFD2* and *DHR2* expression after 30’ following addition of glucose was severely inhibited in *tap42-11* cells (Fig. 6, Additional file 3: Fig. S9 and Additional file 2: Table S1). Furthermore, addition of rapamycin had a reduced effect on *GFD2* and *DHR2* expression in *tap42-11* cells in comparison to wild type cells suggesting that TORC1 regulates *GFD2* and *DHR2* expression mainly via Tap42. For the remaining 5 TGC genes, addition of glucose affected their expression to comparable extents in both wild type and *tap42-ts* strains (Fig. 6, Additional file 2: Table S1 and Additional file 3: Fig. S9). Interestingly, addition of rapamycin had little or no effect on expression of *GPG1*, *UGA1*, *RME1*, and *CIT1* genes in *tap42-ts* cells (Fig. 6, Additional file 2: Table S1 and Additional file 3: Fig. S9). In addition, the effect of rapamycin on *CRC1* expression in *tap42-11* cells was reduced by 3-10-fold at 120’ in comparison to wild type cells (Additional file 2: Table S1). These results indicate that TORC1 regulates glucose-driven changes in the TGC genes through Tap42.

### Rrd1/Rrd2 and Sit4 proteins are required for TORC1’s role in the transcriptional response to glucose

**Fig. 7:**
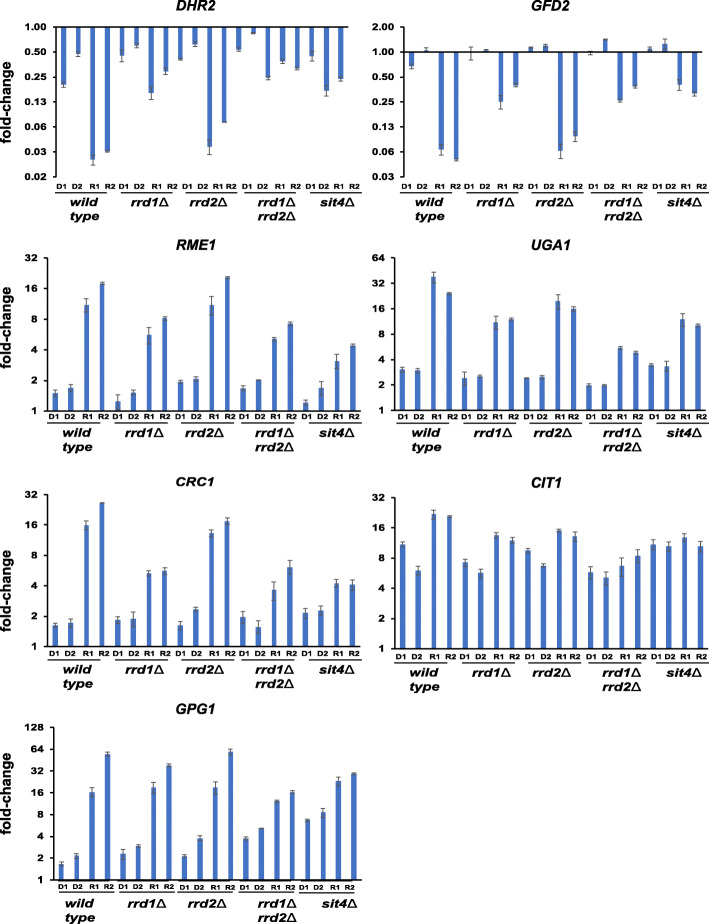
TORC1 regulates the glucose-responsive genes via Sit4 and Rrd1/Rrd2 proteins. Wild type, *sit4Δ*, *rrd1Δ*, *rrd2Δ*, and *rrd1Δ rrd2Δ* cells were grown to logarithmic phase in YPD medium and then treated with either rapamycin (200 nM) or DMSO. Aliquots of the cultures were taken after 0’, 30’, and 60’. RNA was extracted from the cultures, and the expression of the indicated 7 TGC genes was analyzed by real-time qRT-PCR. D1/R1 and D2/R2 indicate DMSO-treated/rapamycin-treated cells after 30’ and 60’ respectively, and the expression fold-change values were normalized with respect to DMSO-treated cells at *t* = 0’. Data are presented as means ± standard deviation (*n* = 2 technical replicates). Data from two additional biological replicates of this experiment are presented in Fig. S10

Tap42 interacts with the catalytic subunit of PP2A phosphatases (PP2Ac) and PP2A-like phosphatase (Sit4) in log-phase cells but not stationary phase cells and keeps them inactive and localized to the vacuole [13]. Upon starvation (or rapamycin treatment), the phosphatases dissociate from Tap42 and dephosphorylate their target proteins in collaboration with two phosphotyrosyl phosphatase activator (PTPA) proteins Rrd1 and Rrd2 [14]. We tested whether the TORC1-mediated regulation of TGC genes is dependent on PP2A-like phosphatase Sit4, Rrd1, and Rrd2. As *sit4Δ* and *rrd1Δ rrd2Δ* strains cannot grow in the presence of glycerol as a carbon source, we tested the roles of Sit4 and Rrd1/2 in the maintenance of TGC gene expression. We treated wild type, *sit4*Δ, *rrd1*Δ, *rrd2*Δ, and *rrd1*Δ *rrd2*Δ cells growing in YPD (with 2% glucose) medium with either DMSO or rapamycin and assessed the expression of TGC genes by real-time qRT-PCR. As expected, rapamycin treatment decreased the expression of *GFD2* and *DHR2* genes and increased the expression of *CIT1*, *CRC1*, *UGA1*, *RME1*, and *GPG1* genes in wild type cells. However, rapamycin-induced changes in expression of TGC genes were reduced considerably in *rrd1Δ rrd2Δ* and *sit4Δ* cells (Fig. 7, Additional file 2: Table S1 and Additional file 3: Fig. S10 ). Rapamycin-induced changes in TGC gene expression were slightly reduced in the *rrd1*Δ strain but unaffected in the *rrd2*Δ strain. However, the rapamycin-induced changes in the *rrd1*Δ *rrd2*Δ strain were further reduced compared to the *rrd1Δ* strain (Fig. 7, Additional file 2: Table S1 and Additional file 3: Fig. S10) suggesting that Rrd1 and Rrd2 proteins play overlapping roles in TORC1-mediated regulation of TGC genes. Taken together, our results are consistent with the hypothesis that TORC1 regulates the glucose-responsive genes by inhibiting the activities of PP2A/Sit4/Rrd1/Rrd2 phosphatases via Tap42.

To understand how Tap42/PP2A/Sit4 module regulates the transcriptional response to glucose, we evaluated the roles of *RTG1*, *NNK1*, *GAT1*, and *GLN3* that are targets of the Tap42/PP2A pathway in yeast [15]. *RTG1* is a member of the basic helix-loop-helix–leucine zipper (bHLH/Zip) family of transcription factors that is required for activation of the retrograde response pathway [16]. Rtg1 localizes to the nucleus in the presence of a poor nitrogen source [16]**.** Activation of *RTG1* target genes requires Tap42 and Sit4 function [17]. Gln3 and Gat1 belong to GATA family of zinc-finger transcriptional activators that localize to the cytoplasm under nitrogen-rich conditions. TORC1 favors the cytoplasmic retention of Gln3 and Gat1 by promoting their phosphorylation and interaction with a cytosolic protein called Ure2. Under nitrogen-limiting conditions, TORC1 activity is reduced leading to activation of Sit4. Gln3 and Gat1 are dephosphorylated by Sit4 resulting in dissociation from Ure2 and translocation to the nucleus and expression of nitrogen catabolite repression (NCR) genes [18]. Nnk1 is a protein kinase that physically interacts with TORC1 and Ure2 and overexpression of Nnk1 results in constitutive targeting of Gln3 to the nucleus [19].

We treated wild type, *rtg1Δ*, *nnk1Δ*, *gat1*Δ, *gln3*Δ, and *rrd1Δ rrd2Δ* cells growing in YPD (with 2% glucose) with either DMSO or rapamycin and assessed the expression of TGC genes by real-time qRT-PCR. As expected, rapamycin-induced changes in TGC gene expression were severely reduced in *rrd1*Δ *rrd2*Δ cells in comparison to wild type cells (Additional file 3: Fig. S11). TORC1-regulated expression of *UGA1* was inhibited in *gat1Δ* and *gln3Δ* cells (Additional file 3: Fig. S11). TORC1-regulated expression of *CRC1* was reduced in *rtg1Δ* cells*. nnk1Δ* had no effect on TORC1-regulated expression of TGC genes. These results suggested that Gat1, Gln3, and Rtg1 could regulate a subset of glucose-responsive genes.

To test if Gln3, Gat1, and Rtg1 are directly involved in the glucose response, we examined their localization in SC-EG medium and upon addition of glucose (2%) to SC-EG medium. GFP-tagged Gln3, Gat1, and Rtg1 cells were grown to logarithmic phase in SC-URA/EG medium and then glucose (2% final concentration) was added to the cultures in the presence of either rapamycin (200 nM) or DMSO. Nuclear and cytoplasmic distribution of GFP-tagged Gln3, Gat1, and Rtg1 was determined by fluorescence microscopy. A small proportion of cells (~ 10%) in SC-URA/EG medium contained nuclear/nucleocytoplasmic staining of Rtg1. However, Gln3, Gat1, and Rtg1 were cytoplasmic in the majority of cells growing in SC-URA/EG medium and remained cytoplasmic upon addition of the glucose to the cultures (Additional file 3: Fig. S12). In contrast, Gln3, Gat1, and Rtg1 localized to the nucleus upon rapamycin treatment (Additional file 3: Fig. S12). Growth of yeast cells in the absence of glucose and with ammonia as the nitrogen source has been shown to trigger weak nitrogen stress [20] which could account for a few cells with nuclear Rtg1 staining in SC-URA/EG grown cultures (Additional file 3: Fig. S12). Taken together, these results indicate that Gln3, Gat1, and Rtg1 do not directly regulate the transcriptional response to glucose. The observed effect of deleting the 3 transcription factor genes on expression of a subset of glucose-responsive genes (Additional file 3: Fig. S11) is indirect.

### TORC1 is activated during spore germination

To test the physiological importance of TORC1’s role in glucose signaling, we investigated the function of TORC1 in spore germination. Return of spores into vegetative growth cycle upon their transfer to favorable nutrient medium is referred to as spore germination. Glucose (or a fermentable carbon source) is essential for efficient spore germination [21]. Glucose alone is sufficient to trigger spore germination [21]. Moreover, spores are only responsive to glucose during the early stages of germination. Consistent with this, transcriptional changes induced upon transfer of spores into rich medium and glucose are strikingly similar during the initial stages of spore germination [1, 22].

**Fig. 8:**
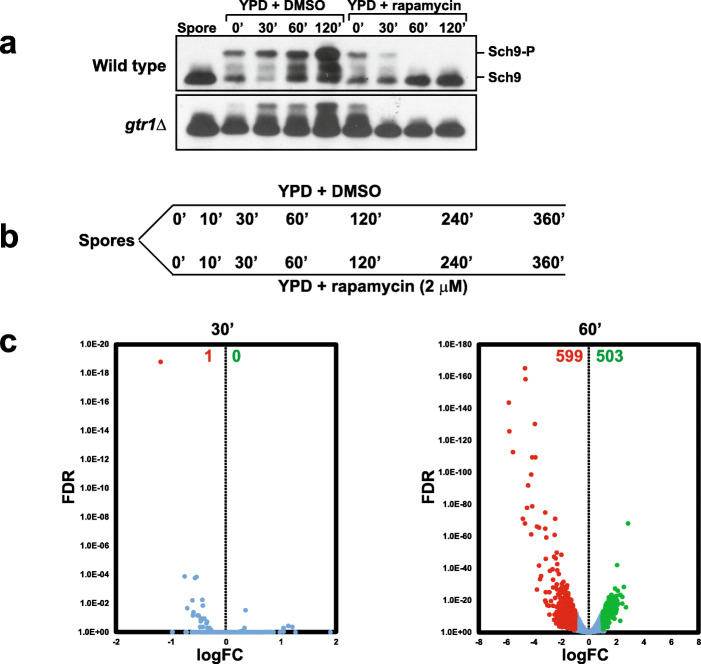
TORC1 regulates transcriptomic changes during spore germination. **a** Wild type spores and *gtr1Δ* spores were transferred into YPD medium in the presence of either DMSO or rapamycin (2 μM). Activity of TORC1 was assayed by monitoring Sch9 phosphorylation using an anti-HA antibody. **b** Experimental outline to analyze the role of TORC1 in spore germination by RNA-Seq. Spores were transferred to YPD medium containing either DMSO or rapamycin (2 μM). Aliquots of yeast cells were taken at the indicated time points (0’ 10’, 30’, 60’, 120’, 240’, and 360’) from the two cultures and used for preparing RNA for RNA-Seq analysis. **c** Transcriptomes of spores treated with rapamycin (2 μM) after 30’ and 60’ were compared with the transcriptomes of corresponding DMSO-treated spores. logFC (fold-change) was plotted against false discovery rate (FDR). Differentially expressed genes (at least 2-fold difference in comparison to DMSO-treated spores) were identified. Genes positively regulated and negatively regulated by TORC1 are indicated by green and red dots respectively and their numbers are indicated at the top of the plot

We first tested whether TORC1 is activated upon transfer of spores into nutrient medium. We transferred stationary phase wild type and *gtr1*Δ diploid cells expressing HA-tagged Sch9 pre-grown in nutrient medium (YPD) grown for 16–20 h into sporulation medium. After 84 h of incubation at 30 °C in sporulation medium, more than 90% of diploid cells had sporulated. We purified wild type and *gtr1*Δ spores and transferred them into nutrient medium (YPD) in the presence of rapamycin (2 μM) or DMSO. Sch9 was completely dephosphorylated in spores indicating that TORC1 is inactive in the spore (Fig. 8a). Sch9 was phosphorylated immediately after transfer of wild type spores into nutrient medium but with a delay in *gtr1*Δ spores (Fig. 8a). Addition of rapamycin to the nutrient medium inhibited Sch9 phosphorylation in both wild type and *gtr1Δ* cells (Fig. 8a) indicating that TORC1 is activated following transfer of spores into nutrient medium.

### TORC1 regulates the glucose-responsive genes during spore germination

To test whether TORC1 regulates the glucose-responsive genes during spore germination, we performed transcriptomic analysis of germinating spores transferred to nutrient medium in the presence and absence of rapamycin. In addition to purified spores, we collected germinating yeast spores at different time points (0’, 10’, 30’, 60’, 120’, 240’ and 360’) following their transfer into nutrient medium (in the presence and absence of rapamycin) and analyzed their transcriptomes by RNA-Seq (Fig. 8b). Our RNA-Seq results are largely consistent with previous transcriptomic analyses of spore germination [1, 22]. Ten classes of genes namely protein synthesis, rRNA processing, gluconeogenesis, TCA sub-cycle, stress, oxidative phosphorylation, proteasome subunits, mating, and cell cycle G1 and cell cycle G2/M were reported to be differentially expressed during spore germination [1] (Additional file 4: Table S2). We found that the aforementioned 10 classes of genes were also differentially expressed following the onset of germination in our experiment (Additional file 5: Fig. S13, Additional file 6: Table S3 and Additional file 7: Table S4).

To determine whether TORC1 regulates the transcriptome during spore germination, we compared the transcriptomes of DMSO- and rapamycin-treated spore germination cultures (Additional file 8: Table S5). There was no significant difference between the transcriptomes at *t* = 30’ between the two cultures (Fig. 8c and Additional file 8: Table S5). However, a significant difference in gene expression between the two cultures was observed after 1 h following transfer of spores into rich medium (Fig. 8c). Around 1102 genes (503 upregulated and 599 downregulated) were found to be differentially expressed (by at least 2-fold) between the two cultures (Fig. 8c and Additional file 8: Table S5). This timing is consistent with our observation that TORC1 activity as measured by Sch9 phosphorylation is inhibited by rapamycin after 1 h following transfer of spores into nutrient medium (Fig. 8c).

We compared our list of 1102 TORC1 targets with the list of TORC1 targets obtained from a microarray analysis-based study [23] and a RNA-Seq study [9] both performed with vegetative cells. Only 90 (18%) of the TORC1 targets identified in the microarray-based study [23] were in our set (Additional file 9: Fig. S14). However, about 935 (84.8%) of the TORC1 targets identified by RNA-Seq [9] were in our target list (Additional file 9: Fig. S14) indicating that the targets of TORC1 are largely similar during spore germination and vegetative growth. We do not know the reason for differences in the TORC1 targets predicted by microarray and RNA-Seq methods but the differences in sensitivities of the two transcriptomic approaches [24] could be a contributing factor.

Expression of the 7 TGC genes during spore germination was affected by rapamycin treatment (Additional file 8: Table S5). To confirm the RNA-seq data, we compared the expression of 7 TGC genes in DMSO- and rapamycin-treated germinating spore cultures after 0’, 30’, 60’, and 120’ following transfer to nutrient medium by real-time qRT-PCR. *DHR2* and *GFD2* genes were upregulated following spore germination, and the genes *CIT1*, *GPG1*, *RME1*, *CRC1*, and *UGA1* were downregulated following transfer of spores into nutrient medium (Additional file 9: Fig. S15). These changes in gene expression mirrored the transcriptional changes that occurred when glucose was added to yeast cells growing in medium containing ethanol and glycerol. Importantly, changes in expression of 7 TGC genes were inhibited by addition of rapamycin (Additional file 9: Fig. S15). These results indicate that TORC1 regulates the glucose-responsive genes during spore germination.

### TORC1 is essential for spore germination

**Fig. 9:**
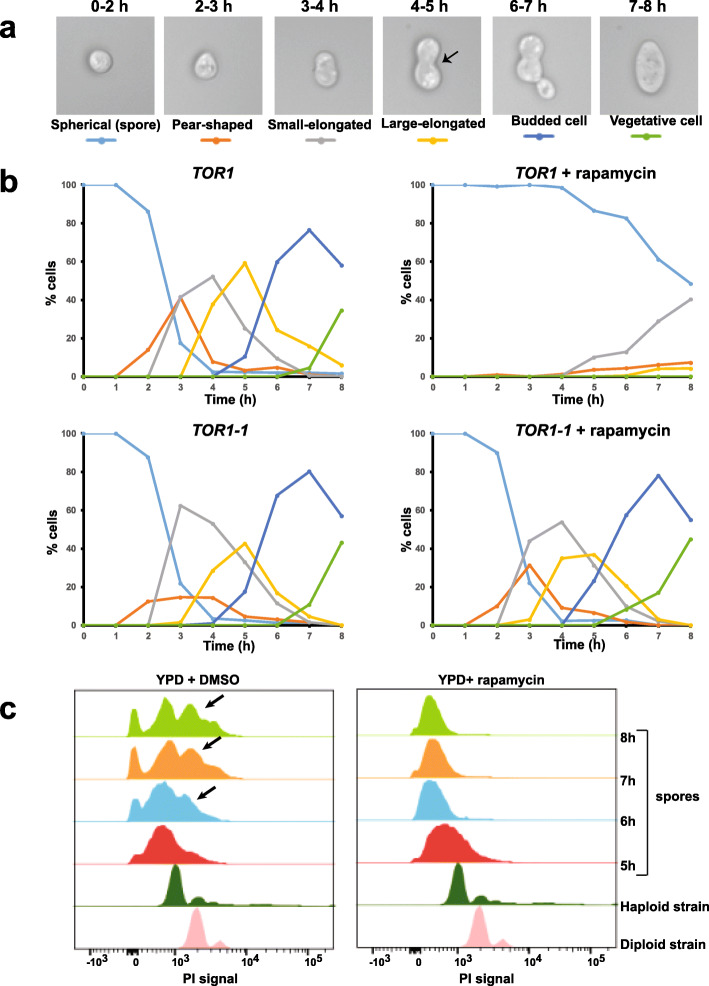
TORC1 activity is required for spore germination. **a** Images showing the morphological changes occurring during spore germination along with their corresponding timing of appearance above. A distinct constriction seen in the germinating spores after 4–5 h following transfer to YPD medium is indicated by the black arrowhead. **b** Wild type and *TOR1-1* spores were transferred into YPD medium in the presence of either DMSO or rapamycin (2 μM). Progress of spore germination was assayed by scoring fraction of cells with different morphologies at the indicated time points for 8 h following transfer into YPD medium. **c** Wild type spores were transferred into nutrient medium in the presence of either DMSO or rapamycin (2 μM). DNA content of germinating spores following 5 h, 6 h, 7 h, and 8 h following transfer to YPD medium was assayed by flow cytometry. Propidium iodide signals of a haploid and diploid strain are indicated for reference

Glucose is essential for efficient germination of yeast spores [21]. As TORC1 regulates the glucose-responsive genes during spore germination, we tested whether TORC1 is required for spore germination. We transferred wild type spores into nutrient medium in the presence of either rapamycin (2 μM) or DMSO (mock-treated) and followed the kinetics of spore germination by bright-field microscopy (Fig. 9a, b). After 2–3 h following transfer into nutrient medium, spores increased in volume and adopted a pear-shaped appearance (Fig. 9a, b). The spores elongated further and displayed a distinct constriction after 4 h. The bud emerged from the mother cell subsequently and detached from the mother cell resulting in the first mitotic division after about 7–8 h following transfer to nutrient medium (Fig. 9a, b). However, in the presence of rapamycin, spore germination was severely inhibited. After 6 to 8 h following rapamycin treatment, 50% of spores had become enlarged but they did not form a constriction seen in untreated cultures and failed to undergo the first mitotic division (Fig. 9a, b). To test whether the effect of rapamycin on spore germination is due to inhibition of the TORC1 complex, we introduced the *TOR1-1* mutation that confers resistance to rapamycin [8]. Rapamycin had no effect on germination of *TOR1-1* spores indicating that the effect of rapamycin on spore germination was due to specific inhibition of TORC1 (Fig. 9b). In contrast to wild type spores, both *fpr1Δ* and *TOR1-1* spores germinated efficiently in the presence of rapamycin (Additional file 9: Fig. S16).

We tested whether TORC1 is required for DNA replication, an event that occurs during final stages of spore germination [1]. We transferred purified wild type spores into nutrient medium in presence and absence of rapamycin and monitored DNA replication by flow cytometry. The intensity of propidium iodide (PI) staining of spores was lower than that of mitotically growing haploid cells presumably because of reduced permeability to PI caused by the presence of spore wall. However, the wild type spores replicated their DNA between 6 and 8 h as indicated by the appearance of cells with increased PI signals. In contrast, the PI signals of rapamycin-treated spores remained unchanged during the course of the experiment indicating that they did not undergo DNA replication (Fig. 9c). We also confirmed that the expression of G1 cell cycle genes (*CLN1*, *CLN2*, *CLB6*, *EGT2*, *PCL1*, *PCL9*, *CHS2*, and *CTS1*) expressed after 4 h of germination was inhibited by rapamycin (Additional file 9: Fig. S17). Taken together, our data indicate that TORC1 is required for spore germination in budding yeast.

### Effect of TORC1 inhibition on spore germination is dependent on Rrd1

If TORC1’s role in regulating the glucose-responsive genes is essential for spore germination, then inactivating the Tap42/Sit4/Rrd1/Rrd2 branch should allow spores germinate even in the presence of rapamycin. As *tap42-11*, *rrd1Δ rrd2Δ*, and *sit4Δ* mutants failed to sporulate, we tested the effect of rapamycin treatment on germination of *rrd1Δ* spores. We took wild type and *rrd1Δ* spores and transferred them to nutrient medium in the presence and absence of rapamycin. Interestingly, *rrd1Δ* spores germinated earlier in comparison to wild type spores as indicated by the formation of budded cells (Additional file 9: Fig. S18). While the germination of wild type spores was inhibited by rapamycin treatment, *rrd1Δ* mutant spores were able to form budded cells in the presence of rapamycin (Additional file 9: Fig. S18). Our results demonstrate that TORC1’s role in the regulation of glucose-responsive genes via the Tap42/ Sit4/Rrd1/2 pathway is essential for spore germination.

## Discussion

Target of Rapamycin Complex 1 (TORC1) connects the presence of nutrients or growth factors in the environment with cellular growth and proliferation in eukaryotes. Dysregulation of TORC1 in humans has been associated with cancer, diabetes, and obesity [25]. Although TORC1’s function in coupling amino acid levels with cellular growth has been studied to considerable extent, its role in glucose signaling is poorly understood and even somewhat controversial. Our work has enhanced the understanding of the relationship between glucose and TORC1 signaling in yeast. Firstly, we report that glucose is necessary and sufficient to activate TORC1. Transferring yeast cells from a medium containing glucose carbon to a medium containing a non-fermentable carbon source without changing the nitrogen source drastically reduces TORC1 activity. Secondly, TORC1 is required for the transcriptional response to glucose, and this is accomplished mainly by inhibition of PP2A/PP2A-like phosphatases via its downstream effector Tap42. Finally, we have uncovered the physiological significance of TORC1’s role in glucose response by showing that TORC1 is required for spore germination, a glucose-dependent developmental transition in yeast.

**Fig. 10:**
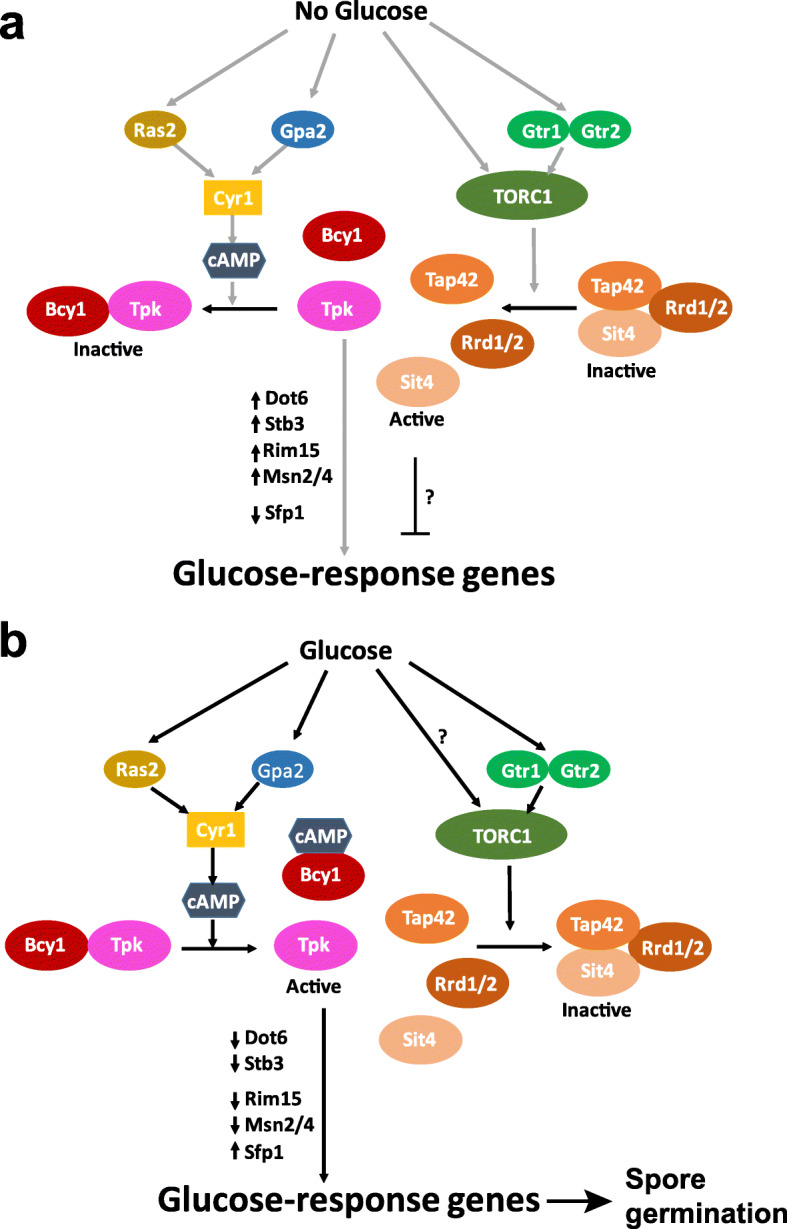
Model for regulation of the transcriptional response to glucose by TORC1 and PKA. Based on the literature and our data, we depict how PKA and TORC1 regulate the glucose response. **a** In the absence of glucose, the PKA and TORC1 pathways are inactive. Bcy1 binds to Tpk1-3 and keeps the protein kinase A inactive. This results in activation of transcriptional repressors Dot6 and Stb3, the protein kinase Rim15 and stress-responsive transcription factors Msn2/Msn4. Sfp1, transcription factor for ribosome biogenesis and protein synthesis genes, is kept inactive. In the absence of TORC1 activity, the PP2A-like protein phosphatase Sit4/Rrd1/2 module dissociates from Tap42 and dephosphorylates unidentified proteins to inhibit the transcriptional response to glucose. **b** In the presence of glucose, the PKA and TORC1 pathways are activated. Ras2 and Gpa2 activate the adenylate cyclase Cyr1 to produce cAMP which binds to Bcy1 and releases Tpk1-3 from Bcy1’s inhibitory effect. PKA inactivates Dot6, Stb3, Rim15, and Msn2/Msn4 and activates Sfp1. TORC1 is activated by glucose through Gtr1/Gtr2-dependent and Gtr1/Gtr2-independent mechanisms. In the presence of active TORC1, Tap42 binds to Sit4/Rrd1/2 module and keeps it inactive thereby preventing its inhibitory effect on the transcriptional response to glucose. Activation of the transcriptional response to glucose is essential for spore germination

Thus far, PKA and TORC1 have been implicated in sensing glucose and nitrogen/amino acid levels respectively in yeast. We propose that TORC1 works with PKA in integrating information from glucose levels with growth and proliferation. A cellular nutrient sensor, in principle, should be activated by the nutrient and should prepare the cell for metabolism of the nutrient. By these two criteria, our study strongly suggests that TORC1 is a glucose sensor in yeast (Fig. 10). Glucose activates TORC1 and TORC1 regulates the glucose-responsive genes (Fig. 10).

A recent study evaluated the contributions of PKA and TORC1 to transcriptional regulation of ribosomal biogenesis and protein synthesis genes following transfer of cells from a non-fermentable carbon source to glucose using chemical inhibitors [26]. They found that while PKA was required for the regulation of ribosomal biogenesis/ protein synthesis genes during transition into glucose-containing medium, TORC1 was required for their steady-state gene expression [26]. Although they proposed that TORC1 regulates the ribosomal biogenesis genes by inactivating transcriptional repressors Dot6 and Tod6 via Sch9, rapamycin treatment affected the gene expression in a *dot6Δ tod6Δ* strain [26]. This is consistent with our observations that rapamycin treatment affected the expression of the TGC genes in the *sch9Δ* strain (Fig. 5 and Additional file 4: Fig. S7). In contrast, rapamycin treatment had no/significantly reduced effect on expression of glucose-responsive genes in strains lacking functional Tap42 or Sit4 or Rrd1/Rrd2 proteins in our experiments. Precisely how PP2A and PP2A-like phosphatase Sit4 regulate the transcription of glucose-responsive genes remains a topic that merits further investigation.

Another key question emerging from our study is how glucose activates TORC1. Glucose deprivation has been reported to cause TORC1 inactivation [7, 15, 27]. However, we show that glucose is also sufficient for TORC1 activation following complete nutrient starvation. This activation is partly dependent on Gtr1/Gtr2 complex but is independent of PKA activity. Although we have suggested that glucose activates TORC1 through Gtr1-dependent and Gtr1-independent pathways, it is formally possible that glucose activates TORC1 through a single pathway and Gtr1/Gtr2 complex regulates the kinetics of TORC1 activation. Snf1 kinase, which is active during glucose starvation, has been shown to inactivate TORC1 by phosphorylating its subunit Kog1 [27]. In addition, glucose has also been reported to regulate TORC1 activity by altering the cytosolic pH which promotes a physical interaction between v-ATPase and Gtr1 [28]. It will be interesting to test whether glucose-induced activation of TORC1 observed in our assay occurs via inhibition of Snf1 and/or activation of v-ATPase.

We found that a higher concentration of rapamycin (2 μM) was required to inhibit TORC1 activity (assayed by Sch9 phosphorylation) in spores and completely starved cells in comparison to vegetative cells growing in SC medium (200 nM). The impermeable spore wall may prevent the uptake of rapamycin into the spore explaining the need for higher concentrations. Higher concentrations of rapamycin were also required for TORC1 inhibition in completely starved cells. We do not know the reason for this observation. One possibility is that formation of higher-order structures of TORC1 complexes like TOROIDs [29] or Kog1 bodies [27] during starvation might render the FRB domain in TOR1/2 less amenable to inhibition by Fpr1-Rapamycin complex. Alternatively, Fpr1 levels or uptake of rapamycin might be reduced in spores and completely starved cells. However, in both cases (spores and completely starved cells), control experiments with *TOR1-1* and *fpr1Δ* strains ruled out any non-specific effect of rapamycin at 2 μM.

Several intriguing questions emerge from our study. Does glucose need to be metabolized by the cell to activate TORC1? Do the mechanisms of TORC1 activation by glucose and amino acids via the Gtr1/Gtr2 complex overlap? What factors regulate the Gtr1-independent pathway of glucose-mediated TORC1 activation? Interestingly in plants, glucose has been shown to activate TORC1 and the TORC1-glucose signaling is essential for transcriptional reprogramming and development [30]. It is quite plausible that the TORC1-glucose signaling is an ancient conserved pathway employed by eukaryotic cells to coordinate presence of nutrients in the environment with their growth and developmental state.

## Conclusions

TORC1 is activated by glucose independently of PKA activity. TORC1 regulates the transcriptional response to glucose via Tap42, Sit4, and Rrd1/2 proteins, and this is essential for reentry of yeast gametes into the vegetative cycle.

## Methods

### Yeast strains and plasmids

All strains are derived from SK1 genetic background. A complete list of yeast strains along with their genotypes can be found in Additional file 10: Table S6.

### TORC1 activity assay

Protein extraction, chemical fragmentation, and Western analysis were performed as previously described with slight modifications [7]. Yeast cells collected at different time points were mixed with tricholoroacetic acid to a final concentration of 6%. Samples were then kept on ice for at least 5 min and further washed twice with cold acetone, and air dried in a fume hood. Lysis of the cells was done in 150 μl of urea buffer (50 mM Tris pH 7.5, 5 mM EDTA, 6 M urea, 1% SDS, 1 mM PMSF, and 1× PPi) with glass beads in a bead beater followed by incubation for 10 min to 65 °C in a thermomixer with shaking at 800 rpm. Then after protein either stored at − 80 °C or performed NTCB cleavage assay. For NTCB cleavage, 30 μl of 0.5 M CHES (pH 10.5) and 20 μl of NTCB (7.5 mM in H_2_O) were added and samples incubated over night at RT with shaking. Then after 1 vol of 2× sample buffer (2× SDS gel-loading buffer, 20 mM TCEP, and 0.5× PPi) was added to the samples. Further proteins were separated on 8% SDS-PAGE gels and transferred onto nitrocellulose membrane. Blots were then blocked with 5% milk in PBS/0.1% Tween 20. Blots were probed with anti-HA antibody 16B12 (1:1000; cat no. 901501, BioLegend) or 3F10 (1:1000; cat no. 11867431001, Roche) followed by sheep anti-mouse antibody (1:5000; ECL^TM^) or goat anti-rat antibody (1:5000; Santa-Cruz Biotechnology) conjugated with horseradish peroxidase. Finally, the blots were developed with ECL prime Western blotting detection reagent (Amersham Pharmacia Biotech).

### Total RNA extraction

Total RNA was isolated from germinating yeast spore cultures and vegetative cells by the “mechanical disruption protocol” using RNeasy MIDI kit (for RNA-Seq) or RNeasy mini kit (for Real-Time qRT-PCR) (Qiagen), following the manufacturer’s instructions. RNA integrity and concentration were assessed using the Bioanalyzer 2100 with the RNA 6000 Nano Lab Chip kit (Agilent) and the ND-1000 UV-visible light spectrophotometer (NanoDrop Technologies).

### RNA-Seq

Poly-A mRNA was enriched from ~ 10 μg of total RNA with oligo dT beads (Invitrogen). ~ 100 ng of poly-A mRNA recovered was used to construct multiplexed strand-specific RNA-Seq libraries as per manufacturer’s instruction (NEXTflexTM Rapid Directional RNA-SEQ Kit, dUTP-Based, v2). Individual library quality was assessed with an Agilent 2100 Bioanalyzer and quantified with a QuBit 2.0 fluorometer before pooling for sequencing on a HiSeq 2000 (1 × 101 bp read) yielding an average read count of 14,009,863 per sample with an average overall alignment: 95.23%. The pooled libraries were quantified using the KAPA quantification kit (KAPA Biosystems) prior to cluster formation. The accession number for the RNA-Seq data reported in this paper is NCBI: GSE110326.

### Differential expression analysis

The raw Fastq reads were trimmed for adapter sequences and low-quality bases using Trimmomatic (v.0.33) [31] (parameters: LEADING:3 TRAILING:3 SLIDINGWINDOW:4:15 MINLEN:36). These trimmed raw reads were then aligned to the yeast genome (*Saccharomyces cerevisiae* strain SK1) using hisat 2[32] (v.2.0.4) (parameters: --rna-strandness R) and the corresponding Ensemble annotation file (Saccharomyces_cerevisiae R64-1-1.86 gtf-file). The resulting sam files were then converted to bam files that were used to generate feature read counts using Python package based htseq-count of HTSeq [33] (v.0.6.1p1) (parameters: default union-counting mode, --stranded = reverse). The HTSeq read counts were used for the differential expression (DE) analysis using the edgeR [34] package (available in R (v.3.1.3)). A false discovery rate (FDR) cutoff of 0.05 and fold-changes were used to sift the genes that were significantly differentially expressed (DE).

### Real-time qRT-PCR

One microgram of total RNA was reverse-transcribed into cDNA using QuantiTect Reverse Transcription Kit (Qiagen)*.* As a control for genomic DNA contamination, the same reaction was performed without Reverse Transcriptase. Real-time PCR was performed in a final volume of 20 μl containing 20 ng of cDNA using SYBR Fast Universal qPCR Kit (Kapa Biosystems) and analyzed using the Quant Studio 6 Flex system (Applied Biosystems). The real-time PCR conditions is one hold at [95 °C, 180 s], followed by 40 cycles of [95 °C, 1 s] and [60 °C, 20 s] steps. After amplification, a melting-curve analysis was done to verify PCR specificity and the absence of primer dimers. For quantification, the abundance of each gene was determined relative to the house-keeping transcript *ACT1* and the relative gene expression between the DMSO control and rapamycin-treated conditions were calculated using the 2^−ΔΔCt^ method [35]. A list of primers used for real-time qRT-PCR is provided in Additional file 10: Table S7.

### Phospho-PKA substrate Western blot analysis

For phospho-PKA substrate Western blots, protein extracts were prepared from trichloroacetic acid -treated cells. Cells were collected and suspended in 250 μl of 10% trichloroacetic acid. Next, the cells were disrupted with glass beads on Precellys® 24 homogenizer (Bertin Technologies). After that, the trichloroacetic acid pellets were resuspended in 100 μl of 2× SDS gel-loading buffer plus 50 μl of 1 M Tris base to adjust the pH. Samples were then boiled for 10 min and followed by centrifugation at 14,000×*g* for 5 min. The supernatants were separated on 8% SDS-PAGE gels and transferred onto nitrocellulose membranes. Blots were then blocked with 5% bovine serum albumin (BSA) in TBS/0.1% Tween 20. For phospho-PKA substrate, blots were probed with phospho-PKA substrate (RRXS*/T*) antibody (1:1000; cat no. 9624, cell signaling technology) followed by goat anti-rabbit antibody conjugated with horseradish peroxidase (1:5000; Santa-Cruz). For β-actin, blots were probed with anti-beta actin antibody (1:5000; cat no. ab8224, Abcam) followed by goat anti-mouse antibody conjugated with horseradish peroxidase (1:5000; Santa-Cruz). Finally, the blots were developed with ECL prime Western blotting detection reagent (Amersham Pharmacia Biotech).

### Fluorescence microscopy of GFP-tagged transcription factors

Plasmids pRS416-Gln3-GFP and pRS416-Gat1-GFP were gifts from Prof. Terrance Cooper (University of Tennessee Health Science Centre) [36]. Plasmid encoding GFP-tagged Rtg1 (pRS416-Rtg1-GFP) was constructed by replacing Gln3 promoter and ORF sequences in pRS416-Gln3-GFP with the Rtg1 promoter (800 bp upstream of start codon) and ORF sequences. Wild type yeast cells containing pRS416-Gln3-GFP or pRS416-Gat1-GFP or pRS416-Gat1-GFP were grown to logarithmic phase in -URA/EG medium and then glucose (2% final concentration) was added to the cultures in the presence of either rapamycin (200 nM) or DMSO. Nuclear and cytoplasmic distribution of GFP-tagged Gln3, Gat1, and Rtg1 was determined by fluorescence microscopy. Experiments were performed in biological duplicates, and 240 cells were counted for each experiment.

### Sporulation

Yeast strains were streak purified on YPD plates (1% yeast extract, 2% peptone, 2% glucose, and 2% agar). At least five colonies were patched onto YPD plates and incubated at 30 °C for 24 h. Cells were then patched onto sporulation plates (0.82% sodium acetate, 0.19% potassium chloride, 0.035% magnesium sulfate, 0.12% sodium chloride, and 1.5% agar) and YPD plates and incubated at 30 °C for 24 h. Sporulation efficiency was examined using a light microscope, and the corresponding YPD patch from the colony that sporulated best was inoculated in 50 ml YPD liquid media (250 ml conical flask). Cells were grown in a 30 °C shaker at 220 rpm for 16 h. Cells were then washed twice with sporulation medium (0.25% yeast extract, 1.5% potassium acetate, 0.25% glucose). Cells (3 OD_600_/ml) were resuspended in 50 ml of liquid sporulation media (500 ml conical flask). Sporulation was induced for three and half days in the 30 °C shaker at 220 rpm.

### Spore purification

Spore purification was carried out as previously described with slight modifications [22]. Briefly, the sporulation culture was centrifuged at 1000 g for 5 min. Cells (40 OD_600_/ml) were resuspended in softening buffer (10 mM dithiothreitol, 100 mM Tris-SO_4_, pH 9.4) and incubated for 15 min with shaking at 30 °C. Cells were pelleted and resuspended in spheroplasting buffer (2.1 M sorbitol, 10 mM potassium phosphate, pH 7.2). Zymolyase-20 T (cat no. 320921, MP Biomedicals, LLC) was added to the cells at a concentration of 2 mg/ml. Spheroplasting reaction was performed for 30 min with shaking at 30 °C. The suspension was washed thrice and resuspended in spheroplasting buffer. The spore suspension was sonicated (amplitude 40%, cycle 10 of 20 s with a 10-s interval) briefly to disperse the spores and kept on ice. Fresh spores were used for all the experiments.

### Spore germination

Purified spores were suspended at approximately 1 OD_600_ cells/ml YPD medium or 2% glucose and incubated at 30 °C with 220 rpm shaking. Samples were taken for analysis at different time points including the zero-time point. To examine the germination under different conditions, purified spores were incubated with chemicals in YPD medium or 2% glucose and sampling was performed over different time points.

### Microscopy

Spore cultures at different time points were fixed by 4% paraformaldehyde for 15 min with shaking at room temperature. Fixed spores were washed and resuspended in 100 mM K-phosphate buffer (pH 7.5) / 2 M sorbitol. Spores were briefly sonicated and directly visualized under either bright-field or fluorescence microscope.

## Supplementary Information

**Additional file 1: Fig S1.** 1-NM-PP1 inhibits the growth and PKA activity in *pka-as* cells but not in wild type cells. a Wild type (PKA) and *pka-as* yeast cultures were diluted to a starting OD=O.2 in YPD medium containing either DMSO or 1-NM-PP1 at the various concentrations indicated and incubated at 30 °C in a shaker (250 rpm). Normalized growth after 24 hours of incubation at 30 °C is plotted for the various cultures. b Wild type and *pka-as* cells were grown to log phase and then either DMSO or 1-NM-PP1 at different concentrations (1.5, 3.12, 6.25, 12.5 and 25 μM) was added to the cultures. Aliquots of the cultures were taken after 0’, 60 and 120’ and used for preparing protein extracts. Protein samples were analyzed by Western blotting using anti-PKA substrate and anti-actin antibodies. **Fig S2.** Glucose-induced TORC1 activation does not require PKA activity. Wild type and *pka-as* cells subjected to complete nutrient starvation were transferred to 2% glucose solution in the presence of either DMSO or rapamycin (2 μM) or 1-NM-PP1 (1.5 μM). Aliquots of the cultures were taken after 0’, 10’, 20’ and 30’ and used for preparing protein extracts. Protein samples were analyzed by Western blotting using anti-PKA substrate and anti-actin antibodies. **Fig S3.** TORC1 and PKA co-regulate the expression of glucose-responsive genes. Wild type (PKA) and *pka-as* cells were grown to logarithmic phase in SC/EG medium and then glucose (2% final concentration) was added to the cultures in the presence of either rapamycin (200 nM) or 1-NM-PP1 (1.5 μM) or DMSO. Aliquots of the cultures were taken after 0’, 30’ and 60’. RNA was extracted from the cultures and the expression of the 7 TGC genes were analyzed by Real-Time qRT-PCR. Data from these two additional biological replicates 2 and 3 are presented as means ± standard deviation (n = 2 technical replicates).

**Additional file 2: Table S1.** Comparison of Real-Time qRT-PCR data from 3 replicates for experiments discussed in Fig. 4 (Tab labelled PKA), Fig. 5 (Tab labelled sch9), Fig. 6 (Tab labelled tap42) and Fig. 7 (Tab labelled rrd1,2 sit4).

**Additional file 3: Fig. S4.** TORC1 is required to maintain the expression of TGC genes in glucose-containing growth medium. Wild type cells grown into mid-log phase in YPD medium were treated with either DMSO or rapamycin (200 nM). Aliquots of the cultures were taken after 0, 0.5, 1 and 2 hours. RNA was extracted from the cultures and the expression of the TGC genes was analyzed by Real-Time qRT-PCR. Data are presented as means ± standard deviation (n = 2 replicates). **Fig. S5.** TORC1 regulates the glucose-responsive genes independently of Bcy1 T129 dephosphorylation. Cells expressing wild type Bcy1 and mutant *bcy1- T129D* were grown to logarithmic phase in SC-EG medium. Glucose was added to the cultures at the final concentration of 2% along with either rapamycin (200 nM) or DMSO. Aliquots of the cultures were taken after 0’, 15’ and 30’. RNA was extracted from the cultures and the expression of the glucose response genes *DHR2*, *CIT1* and *RME1* were analyzed by Real-Time qRT-PCR. Data are presented as means ± standard deviation (n = 2 replicates). **Fig. S6.** Inhibition of TORC1 has no detectable effect on overall PKA activity. Wild type (PKA) and *pka-as* cells were grown to logarithmic phase in YPD medium (2% glucose) and then either DMSO or rapamycin (200 nM) or 1-NM-PP1 (25 μM) was added to the cultures. a Whole cell extracts were prepared from aliquots of cells taken after 0, 1, 2 and 3 h and were analyzed by Western blotting using an anti-PKA substrate antibody and actin antibody (loading control). b RNA was extracted from aliquots of DMSO and rapamycin-treated cells taken after 0, 1, 2 and 3 h and the expression of *DIP5* and *GAP1* was analyzed by Real-Time qRT-PCR. Data are presented as means ± standard deviation (n = 2 technical replicates). **Fig. S7.** TORC1 regulates the expression of glucose-responsive genes independently of Sch9. Wild type and *sch9Δ* cells were grown to logarithmic phase in SC/EG medium and then glucose (2% final concentration) was added to the cultures in the presence of either rapamycin (200 nM) or DMSO. Aliquots of the cultures were taken after 0’, 30’, 60’ and 120’. RNA was extracted from the cultures and the expression of the indicated 7 TGC genes (*GFD2*, *GPG1*, *UGA1*, *RME1*, *CIT1*, *CRC1* and *DHR2*) were analyzed by Real-Time qRT-PCR. Data from these two additional biological replicates 2 and 3 are presented as means ± standard deviation (n = 2 technical replicates). **Fig. S8.** Growth of *sch9Δ* strain is impaired. Wild type and *sch9Δ *cells were diluted to a starting OD=O.2 in SC/D (SC medium containing 2% glucose) and the growth of the cultures was monitored by measuring the OD_600 nm_ of the cultures every 2 hours for 10 hours. Data are presented as means ± standard deviation (n = 2). **Fig. S9.** Regulation of glucose-responsive genes by TORC1 is dependent on Tap42. Wild type or *tap42-11* cells were grown to logarithmic phase at 25 °C (permissive temperature) in SC/EG medium and then shifted to 37 °C for 30’ to inactivate *tap42-11*. Glucose (2% final concentration) was added to the cultures in the presence of either rapamycin (200 nM) or DMSO. Aliquots of the cultures were taken after 0’, 30’, 60’ and 120’. RNA was extracted from the cultures and the expression of the 7 TGC genes (*GFD2*, *GPG1*, *UGA1*, *RME1*, *CIT1*, *CRC1* and *DHR2*) were analyzed by Real-Time qRT-PCR. Data from these two additional biological replicates 2 and 3 are presented as means ± standard deviation (n = 2 technical replicates). **Fig. S10.** TORC1 regulates the glucose-responsive genes by inhibiting Sit4 and Rrd1/Rrd2 proteins. Wild type, *sit4Δ*, *rrd1Δ*, *rrd2Δ*, and *rrd1Δ rrd2Δ* cells were grown to logarithmic phase and then treated with either rapamycin (200 nM) or DMSO. Aliquots of the cultures were taken after 0’, 30’ and 60’. RNA was extracted from the cultures and the expression of the 7 TGC genes (*GFD2*, *GPG1*, *UGA1*, *RME1*, *CIT1*, *CRC1*, *DHR2* and *GPG1*) were analyzed by Real-Time qRT-PCR. D1/R1 and D2/R2 represent samples from DMSO-treated/ Rapamycin-treated cells after 30’ and 60’ respectively and the expression fold-change values were normalized with respect to DMSO-treated cells at t = 0’. Data from these two additional biological replicates 2 and 3 are presented as means ± standard deviation (n = 2 technical replicates). **Fig. S11.** Tap42/PP2A/Sit4 module regulates the transcriptional response to glucose partly via its targets Gln3, Gat1 and Rtg1. Wild type, *rtg1Δ*, *nnk1Δ*, *gat1*Δ, *gln3*Δ and *rrd1Δ rrd2Δ* cells were grown to logarithmic phase in YPD medium and then treated with either rapamycin (200 nM) or DMSO. Aliquots of the cultures were taken after 0’, 30’ and 60’. RNA was extracted from the cultures and the expression of the 7 TGC genes (*GFD2*, *GPG1*, *UGA1*, *RME1*, *CIT1*, *CRC1* and *DHR2*) was analyzed by Real-Time qRT-PCR. 1/2 and 3/4 represent samples from DMSO-treated/ Rapamycin-treated cells after 30’ and 60’ respectively and the expression fold-change values were normalized with respect to DMSO-treated cells at t = 0’. Data are presented as means ± standard deviation (n = 2 technical replicates). **Fig. S12.** Localization of the transcription factors Gln3, Gat1 and Rtg1 is not altered during the glucose response. GFP-tagged Gln3, Gat1 and Rtg1 cells were grown to logarithmic phase in -URA/EG medium and then glucose (2% final concentration) was added to the cultures in the presence of either rapamycin (200 nM) or DMSO. Nuclear and cytoplasmic distribution of GFP-tagged Gln3, Gat1 and Rtg1 was determined by fluorescence microscopy. Experiments were performed in biological duplicates and 240 cells were counted for each experiment. Representative images of cells expressing GFP-tagged transcription factors in SC medium ethanol-glycerol before/after addition of glucose (2%) with /without rapamycin ( 200 nM) are shown. Percentage of cells number was plotted against nuclear, cytoplasmic and nucleocytoplasmic distribution of GFP-tagged transcription factors. Data are presented as means ± standard deviation (n = 2 biological replicates).

**Additional file 4: Table S2.** Lists of genes in the 10 categories reported in a transcriptomic study of spore germination in yeast.

**Additional file 5: Figure S13.** Comparative gene expression analysis of specific gene modules during spore germination in the presence and absence of rapamycin. Expression of genes in 10 specific modules described in an earlier transcriptomic study of spore germination (Additional file 3: Table 2) was examined in our RNA-Seq data. The transcripts levels of genes in 10 modules in spores incubated in either YPD + DMSO or YPD + rapamycin, for 0, 0.17, 0.5, 1, 2, 4 and 6 h was compared with the corresponding level in ungerminated spores. Blue and red bars indicate the fold-change values for ‘YPD + DMSO’ and ‘YPD + rapamycin’ cultures respectively. Comparison of gene expression between ‘spores’ with ‘spores + YPD’ or ‘spores + YPD + rapamycin’ are shown in Additional file 6: Table S3 and Additional file 7: Table S4 respectively.

**Additional file 6: Table S3.** This contains the results of the comparative analyses of transcriptomes of purified spores with germinating spore cultures after 0, 10, 30, 60, 120, 240 and 360-minutes following transfer into YPD in the appropriately labelled worksheets. All worksheets have of the differential transcriptomic analysis in which the values of logFC (FC = fold-change in transcript levels) along with the corresponding logCPM (counts per million), False Discovery Rate (FDR) and P-values are indicated for each gene.

**Additional file 7: Table S4.** Tables 1-7 contain the results of the comparative analyses of transcriptomes of ungerminated spores and spores transferred to ‘YPD + rapamycin’ after 0, 10, 30, 60, 120, 240 and 360 minutes following transfer into YPD in the appropriately labelled worksheets All worksheets have the results of the differential transcriptomic analysis in which the values of logFC (FC = fold-change in transcript levels) along with the corresponding logCPM (counts per million), False Discovery Rate (FDR) and P-values are indicated for each gene.

**Additional file 8: Table S5.** This contain the results of the comparative analyses of transcriptomes of ‘Spores transferred to YPD + DMSO’ and ‘spores transferred to YPD + rapamycin’ after 0, 10, 30, 60, 120, 240 and 360-minutes following transfer into YPD in the appropriately labelled worksheets. All worksheets have of the differential transcriptomic analysis in which the values of logFC (FC = fold-change in transcript levels) along with the corresponding logCPM (counts per million), False Discovery Rate (FDR) and P-values are indicated for each gene.

**Additional file 9: Fig. S14.** Overlap of glucose-responsive genes with TORC1 target genes. a Comparison of our TORC1 target list identified during spore germination with those reported in a Micro-array-based study (Shamji et al.) and an RNA-Seq study (Gowans et al.) [ both performed with vegetative cells. b Comparison of the list of glucose-responsive genes (Wang et al.) with TORC1 target lists identified by our RNA-Seq analysis and an independent RNA-Seq study (Gowans et al.). **Fig. S15.** TORC1 regulates the glucose-responsive genes during spore germination. Spores were transferred to YPD medium with either DMSO or rapamycin (2 μM). Aliquots of yeast cells taken at the indicated time points (0 h, 0.5 h, 1 h, and 2 h) from the two cultures were used for preparing RNA. Expression of 7 TGC genes (*GFD2*, *GPG1*, *UGA1*, *RME1*, *CIT1*, *CRC1* and *DHR2*) was assayed by Real-Time qRT-PCR analysis. Levels of transcripts were normalized with respect to actin mRNA. Data are presented as means ± standard deviation (n = 2 technical replicates). **Fig. S16.** TORC1 is required for spore germination. a Fourteen asci resulting from sporulation of wild type or *fpr1Δ* or *TOR1-1* diploid cells were dissected on YPD + agar plates containing 1.5 μM rapamycin. Fourteen asci from wild type diploid cells were also dissected on a YPD agar plate without rapamycin. Percentage of budded cells was calculated by examining the spore morphology under the dissection microscope after 6 hours following dissection and is indicated in the plot. b Images of the agar plates described above following incubation at 30 °C for 2 days are presented. **Fig. S17.** TORC1 is required for expression of cell cycle genes during spore germination. Spores were transferred to YPD medium with either DMSO or rapamycin (2 μM). Aliquots of yeast cells taken at the indicated time points (0, 4, 6 and 7 h) from the two cultures were used for preparing RNA. Expression of cell cycle genes *CLN1*, *CLN2*, *CLB6*, *EGT2*, *PCL1*, *PCL9*, *CHS2* and *CTS1* was assayed by Real-Time qPCR analysis. Levels of transcripts were normalized with respect to actin mRNA. Data are presented as means ± standard deviation (*n* = 2 replicates). **Fig. S18.** TORC1’s role in spore germination is mediated via inhibition of Rrd1. Wild type and *rrd1Δ* spores were transferred into YPD medium in the presence of either DMSO or rapamycin (2 μM). Progress of spore germination was assayed by scoring fraction of cells with different morphologies at the indicated time points for up to 8 hours following transfer into YPD medium.

**Additional file 10: Table S6.** A list of yeast strains used in the study. **Table S7.** A list of primers used for Real-Time qRT-PCR analyses in the study.

## Data Availability

Datasets and strains used in the current study are available from the corresponding author upon request. RNA-Seq data reported in this paper are publicly available at NCBI (Accession number GSE110326).

## Abbreviations

TORC1: Target Of Rapamycin Complex 1
PKA: Protein Kinase A
TGC: TORC1 Glucose Co-regulated
SC: Synthetic complete
TCA: Tricarboxylic acid
qRT-PCR: Quantitative reverse transcription polymerase chain reaction
