# Supplementary figures and images for: TORC1 regulates the transcriptional response to glucose and developmental cycle via the Tap42-Sit4-Rrd1/2 pathway in Saccharomyces cerevisiae
Source: BMC Biol. 2021 May 6;19:95. doi: 10.1186/s12915-021-01030-3 (PMC8103650; doi:10.1186/s12915-021-01030-3)

**a**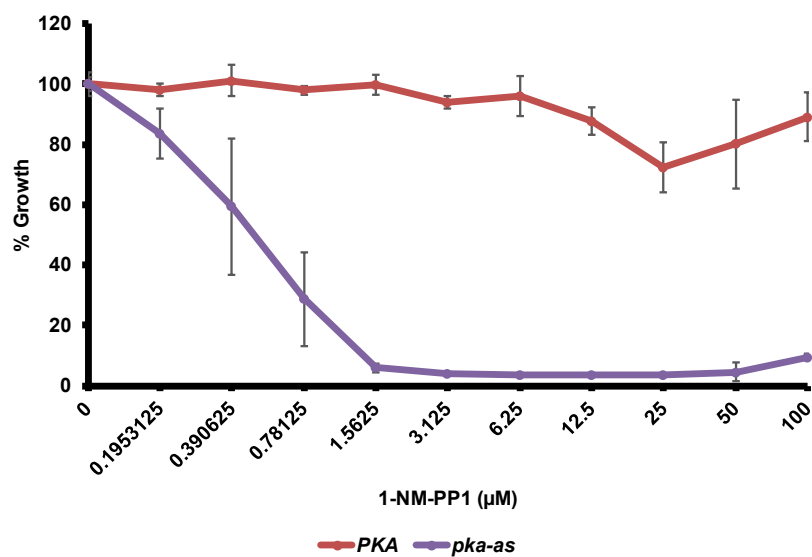**b**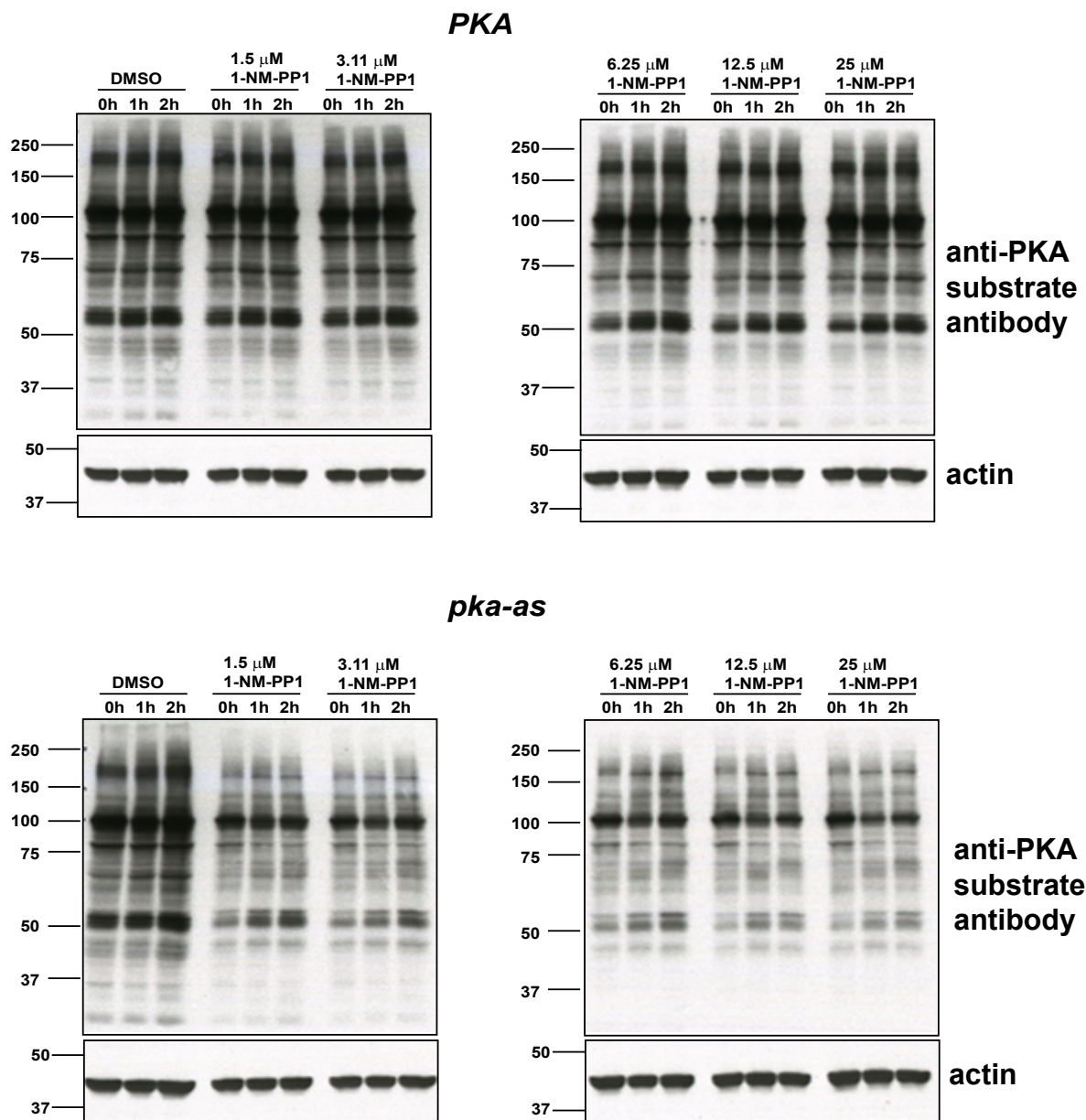**Figure S1**

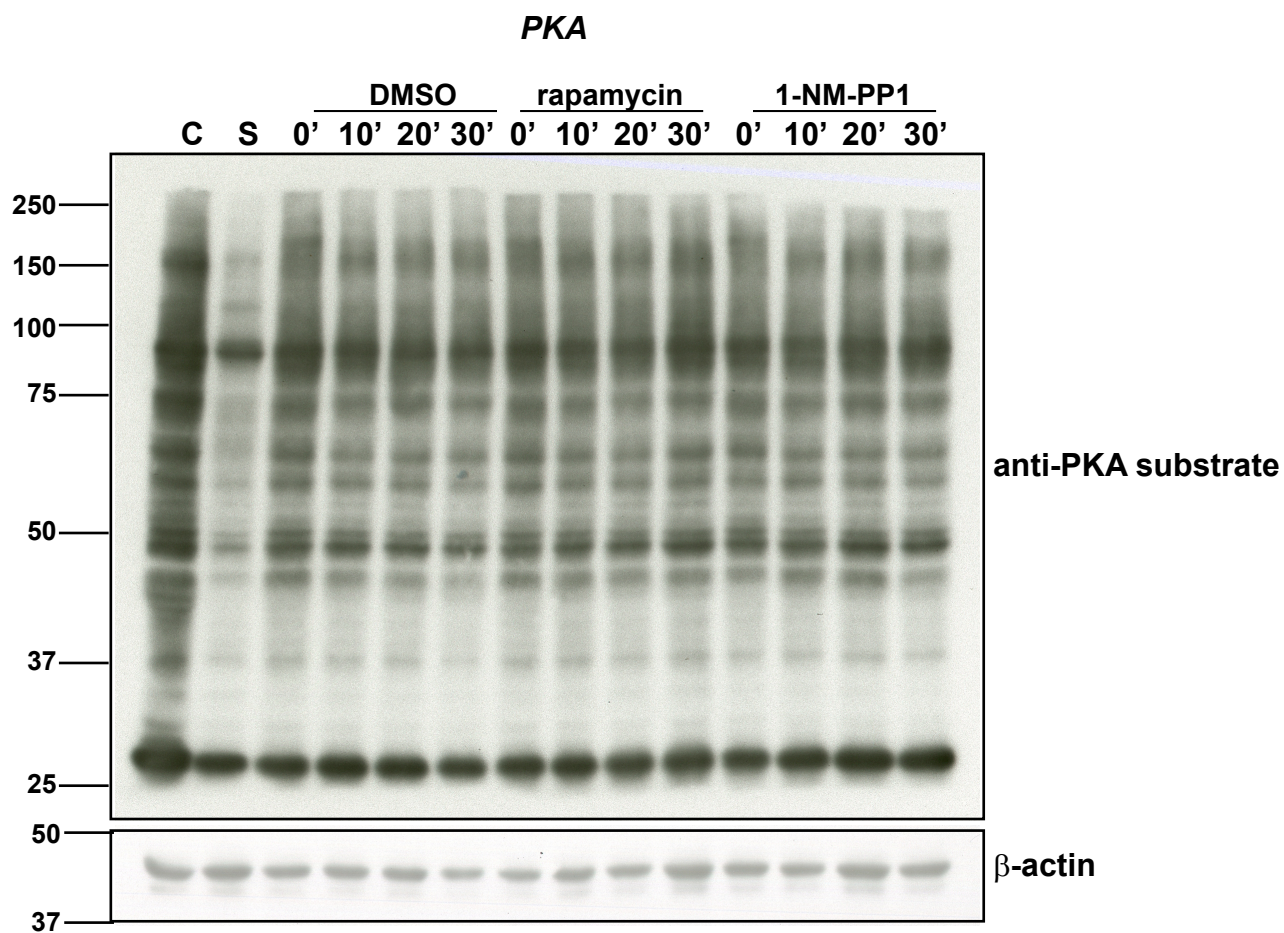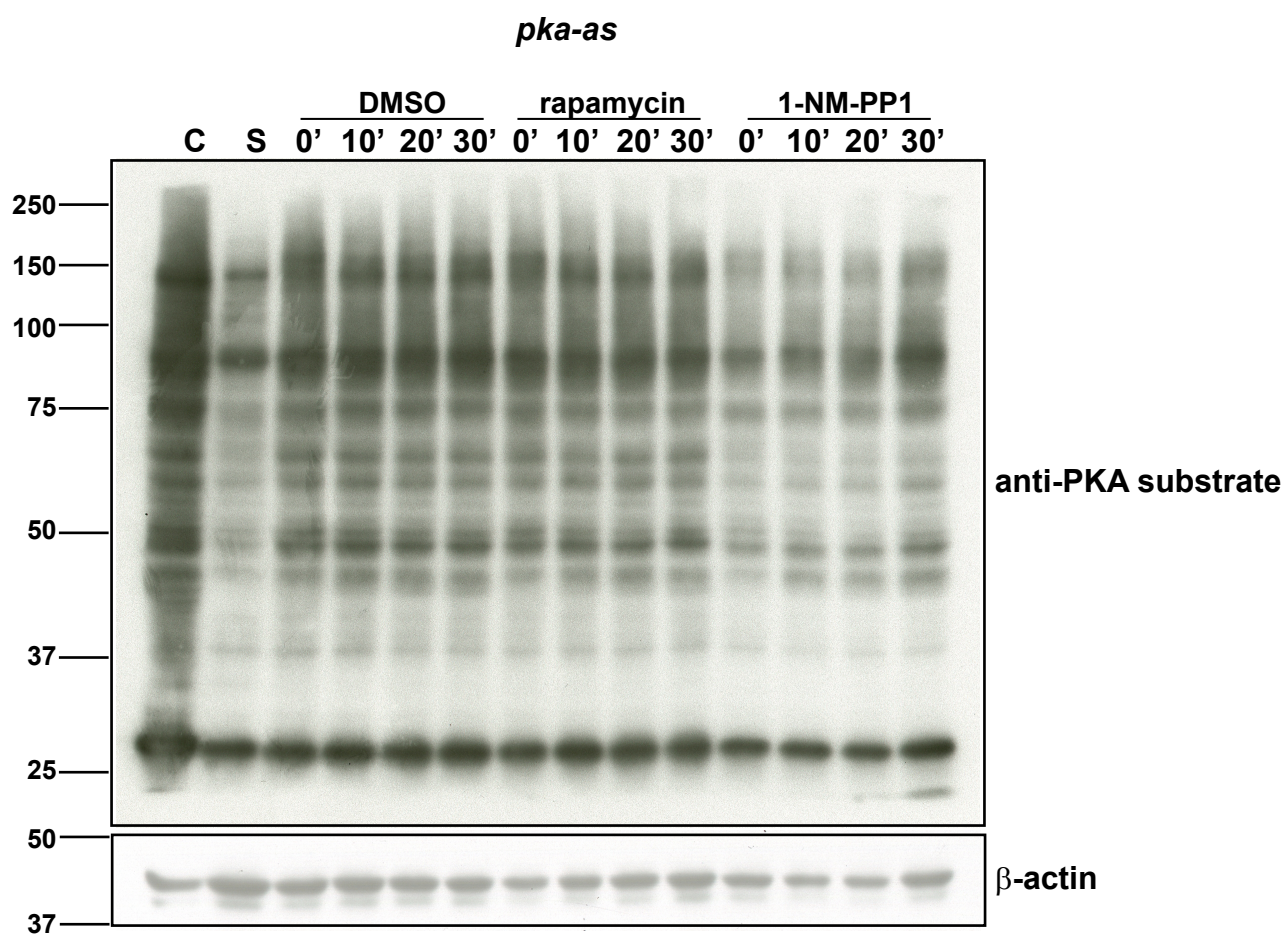

**Figure S2**

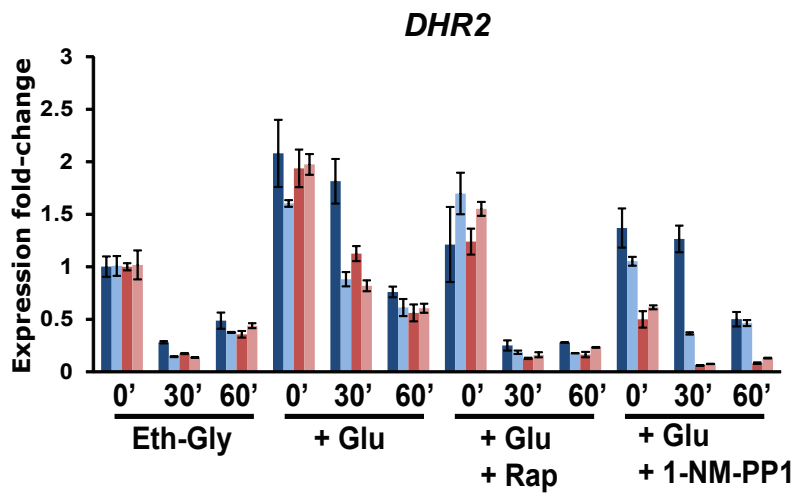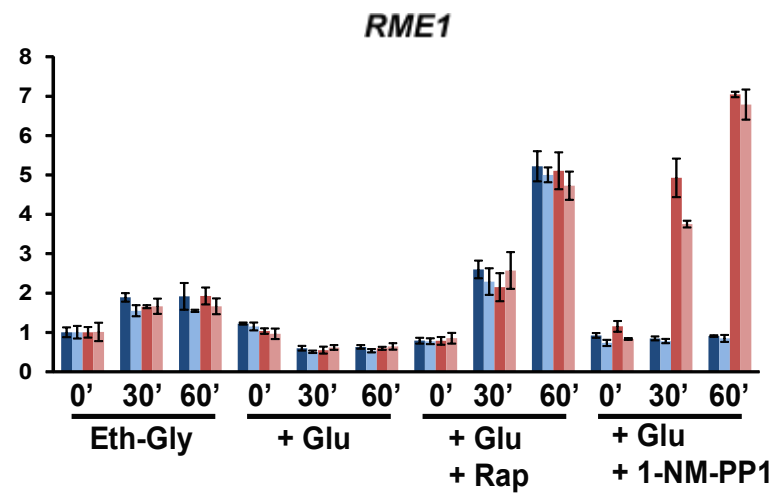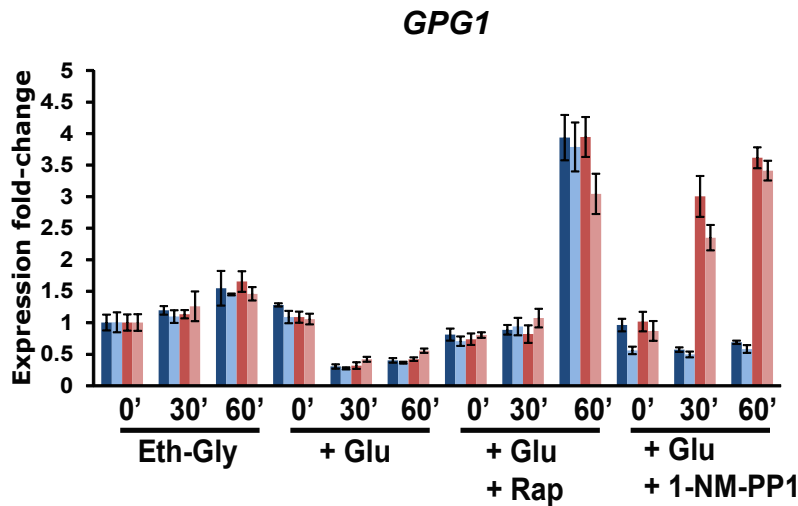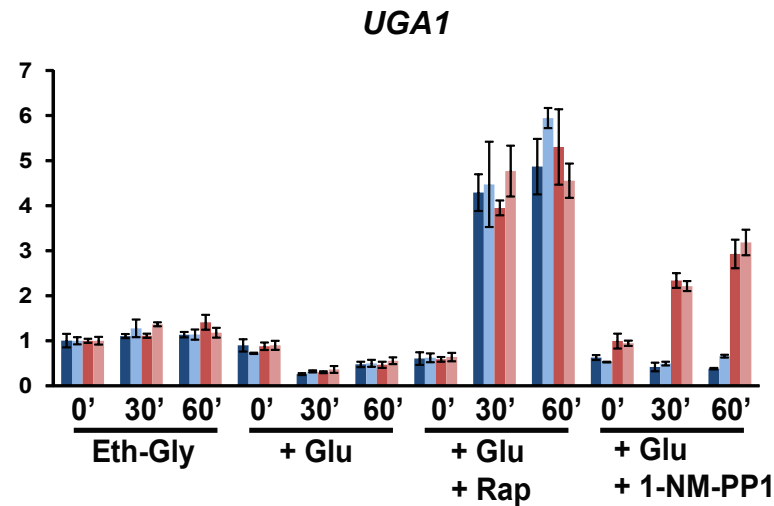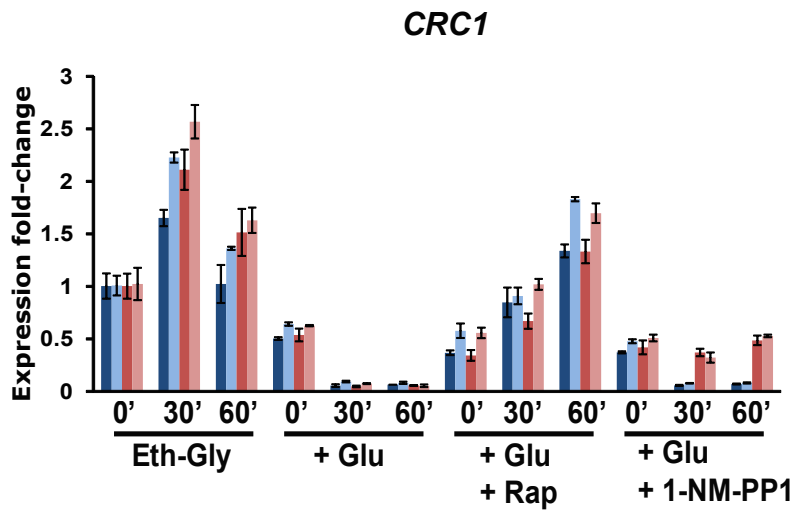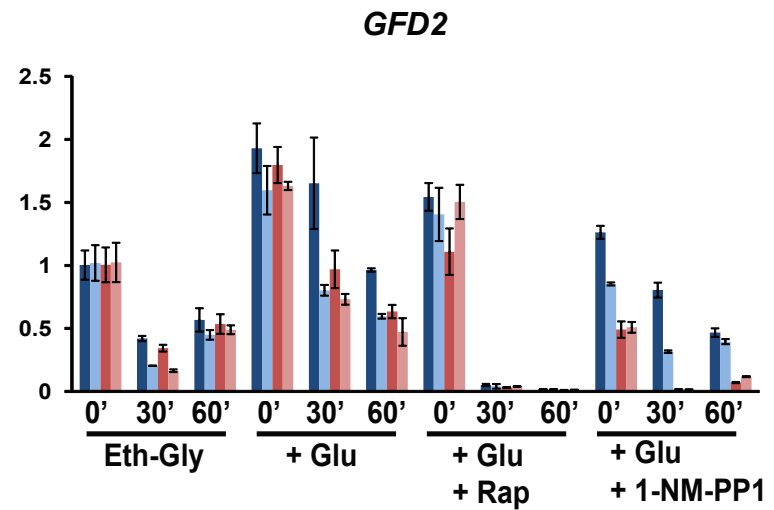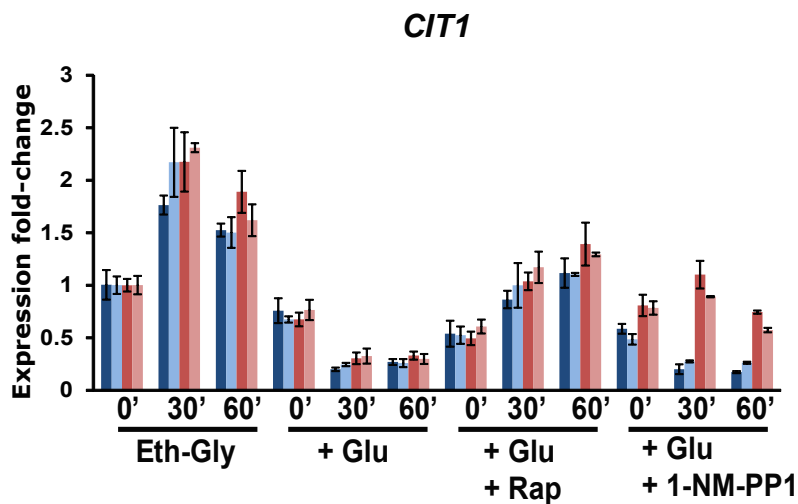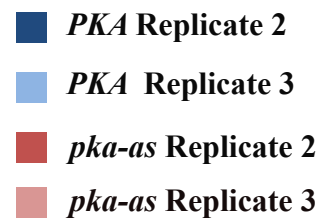

**Figure S3**

Supplement: Supplementary file 1 — Additional file 1: Fig S1. 1-NM-PP1 inhibits the growth and PKA activity in pka-as cells but not in wild type cells. a Wild type (PKA) and pka-as yeast cultures were diluted to a starting OD=O.2 in YPD medium containing either DMSO or 1-NM-PP1 at the various concentrations indicated and incubated at 30 °C in a shaker (250 rpm). Normalized growth after 24 hours of incubation at 30 °C is plotted for the various cultures. b Wild type and pka-as cells were grown to log phase and then either DMSO or 1-NM-PP1 at different concentrations (1.5, 3.12, 6.25, 12.5 and 25 μM) was added to the cultures. Aliquots of the cultures were taken after 0’, 60 and 120’ and used for preparing protein extracts. Protein samples were analyzed by Western blotting using anti-PKA substrate and anti-actin antibodies. Fig S2. Glucose-induced TORC1 activation does not require PKA activity. Wild type and pka-as cells subjected to complete nutrient starvation were transferred to 2% glucose solution in the presence of either DMSO or rapamycin (2 μM) or 1-NM-PP1 (1.5 μM). Aliquots of the cultures were taken after 0’, 10’, 20’ and 30’ and used for preparing protein extracts. Protein samples were analyzed by Western blotting using anti-PKA substrate and anti-actin antibodies. Fig S3. TORC1 and PKA co-regulate the expression of glucose-responsive genes. Wild type (PKA) and pka-as cells were grown to logarithmic phase in SC/EG medium and then glucose (2% final concentration) was added to the cultures in the presence of either rapamycin (200 nM) or 1-NM-PP1 (1.5 μM) or DMSO. Aliquots of the cultures were taken after 0’, 30’ and 60’. RNA was extracted from the cultures and the expression of the 7 TGC genes were analyzed by Real-Time qRT-PCR. Data from these two additional biological replicates 2 and 3 are presented as means ± standard deviation (n = 2 technical replicates). [file 12915_2021_1030_MOESM1_ESM.pdf]

■ (YPD+ DMSO-treated spores) vs (spore)

■ (YPD+ rapamycin-treated spores) vs (spore)

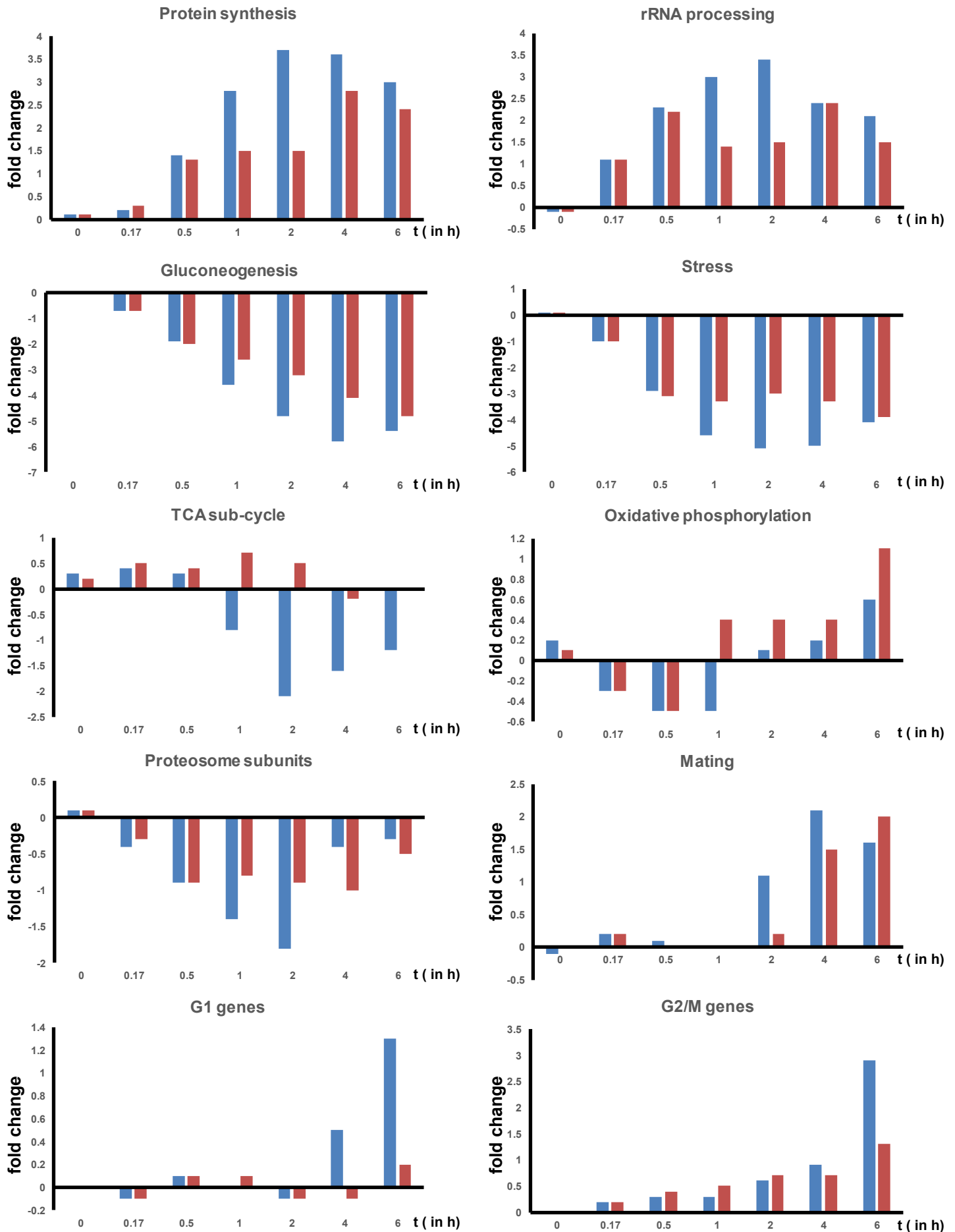

Figure S13

Supplement: Supplementary file 5 — Additional file 5: Figure S13. Comparative gene expression analysis of specific gene modules during spore germination in the presence and absence of rapamycin. Expression of genes in 10 specific modules described in an earlier transcriptomic study of spore germination (Additional file 3: Table 2) was examined in our RNA-Seq data. The transcripts levels of genes in 10 modules in spores incubated in either YPD + DMSO or YPD + rapamycin, for 0, 0.17, 0.5, 1, 2, 4 and 6 h was compared with the corresponding level in ungerminated spores. Blue and red bars indicate the fold-change values for ‘YPD + DMSO’ and ‘YPD + rapamycin’ cultures respectively. Comparison of gene expression between ‘spores’ with ‘spores + YPD’ or ‘spores + YPD + rapamycin’ are shown in Additional file 6: Table S3 and Additional file 7: Table S4 respectively. [file 12915_2021_1030_MOESM5_ESM.pdf]

**a****TORC1 targets**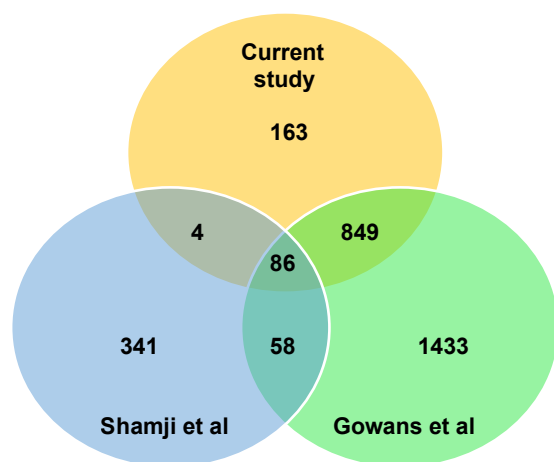**b****Glucose-response genes**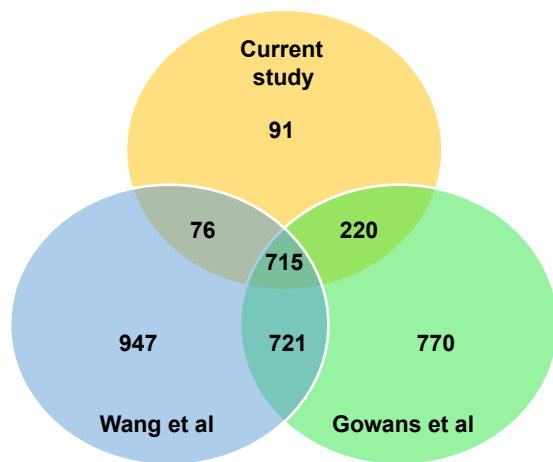**Figure S14**

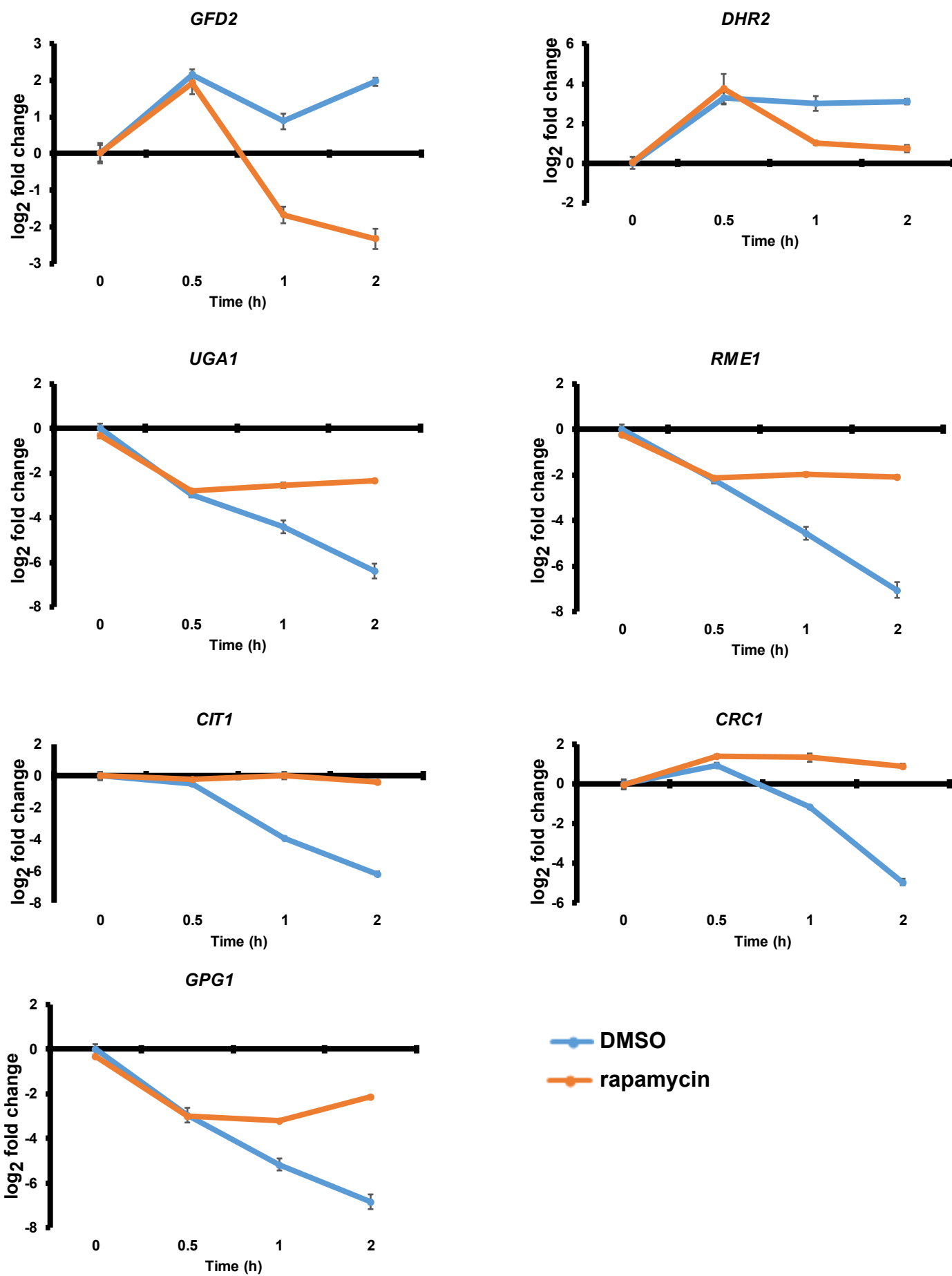

Figure S15

**a**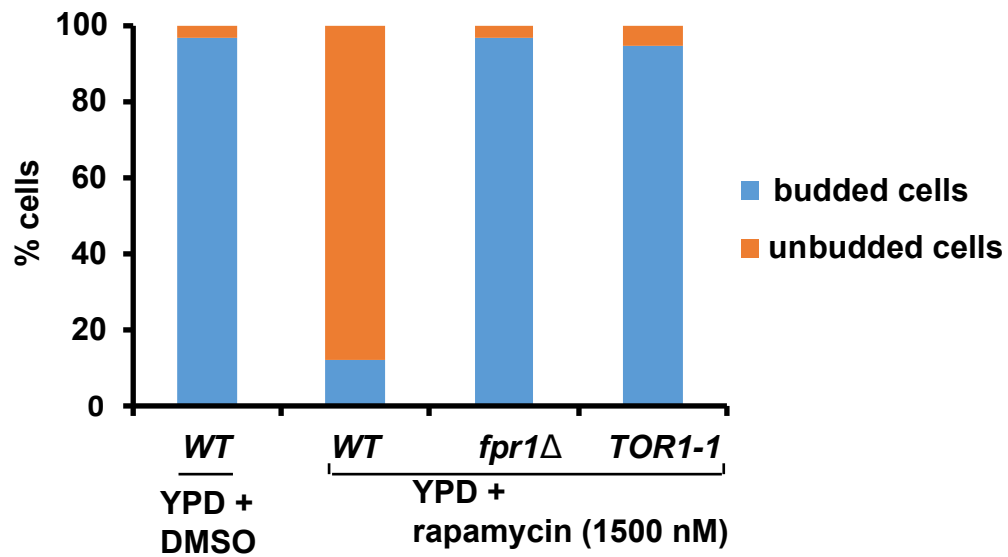**b**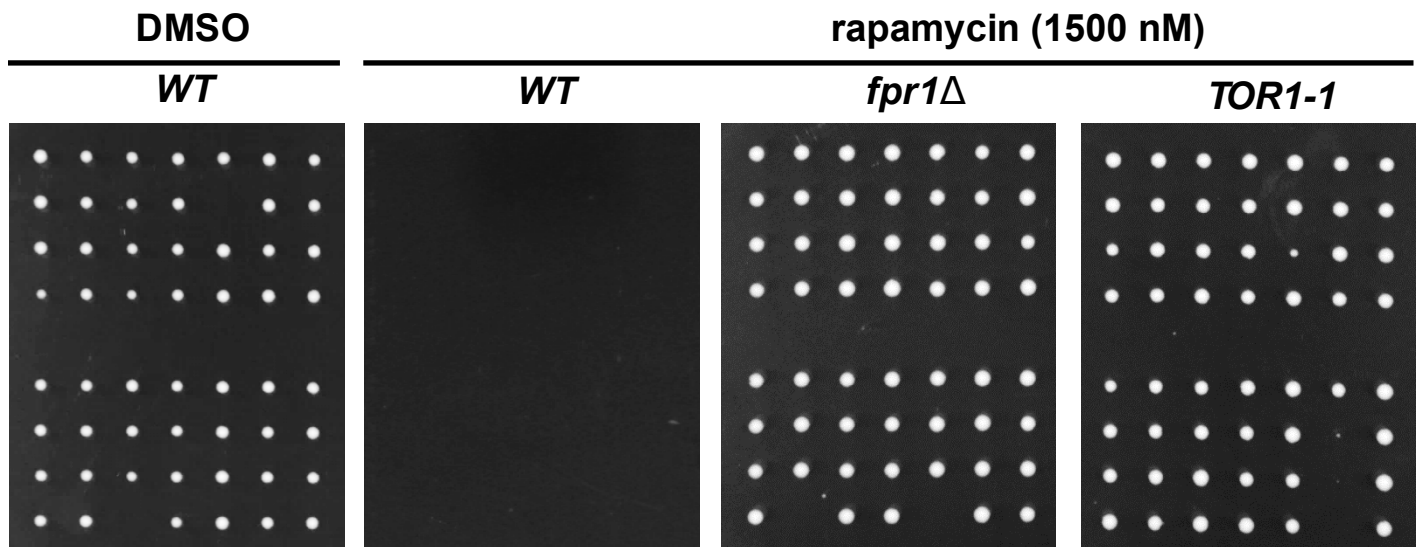**Figure S16**

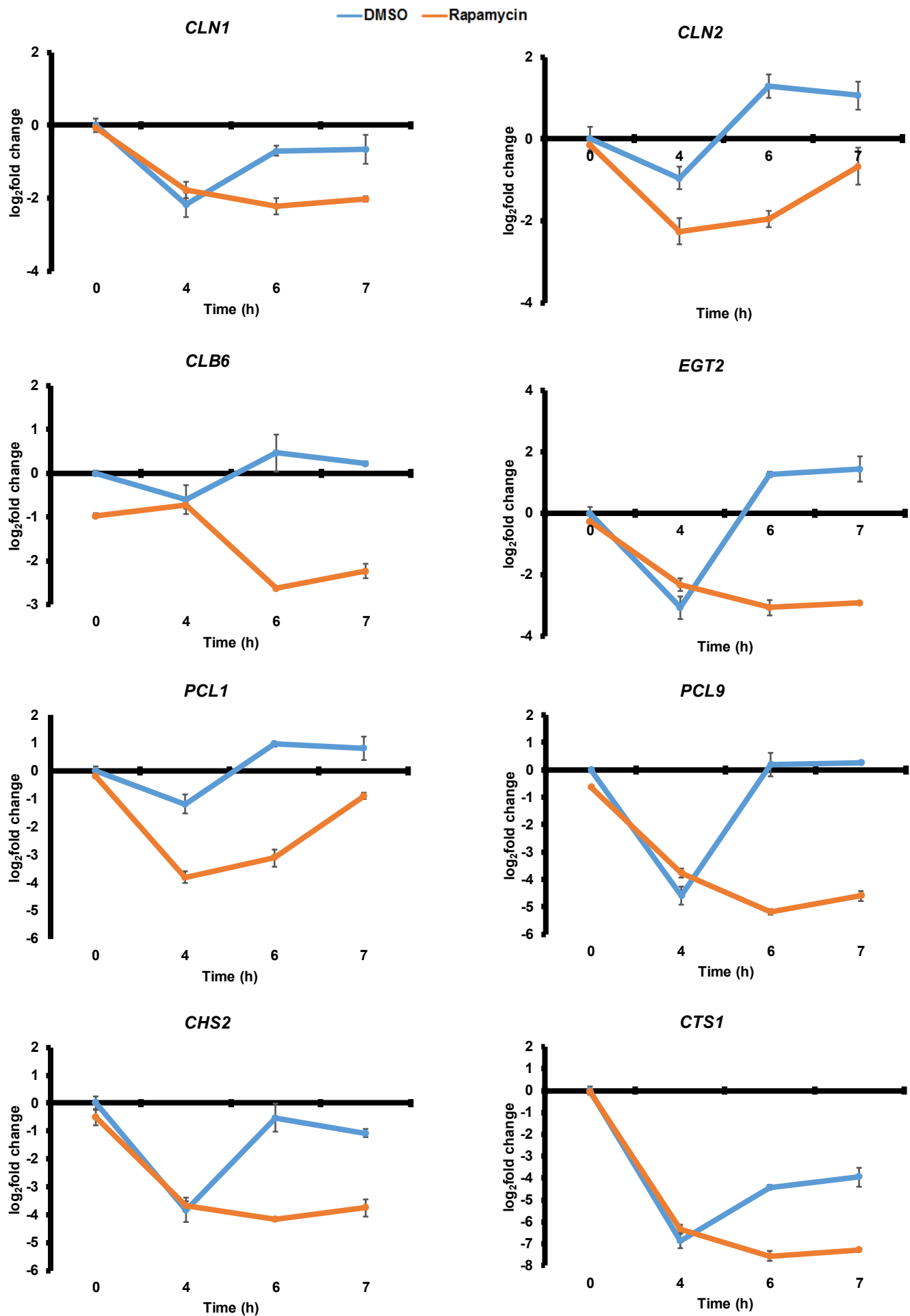

Figure S17

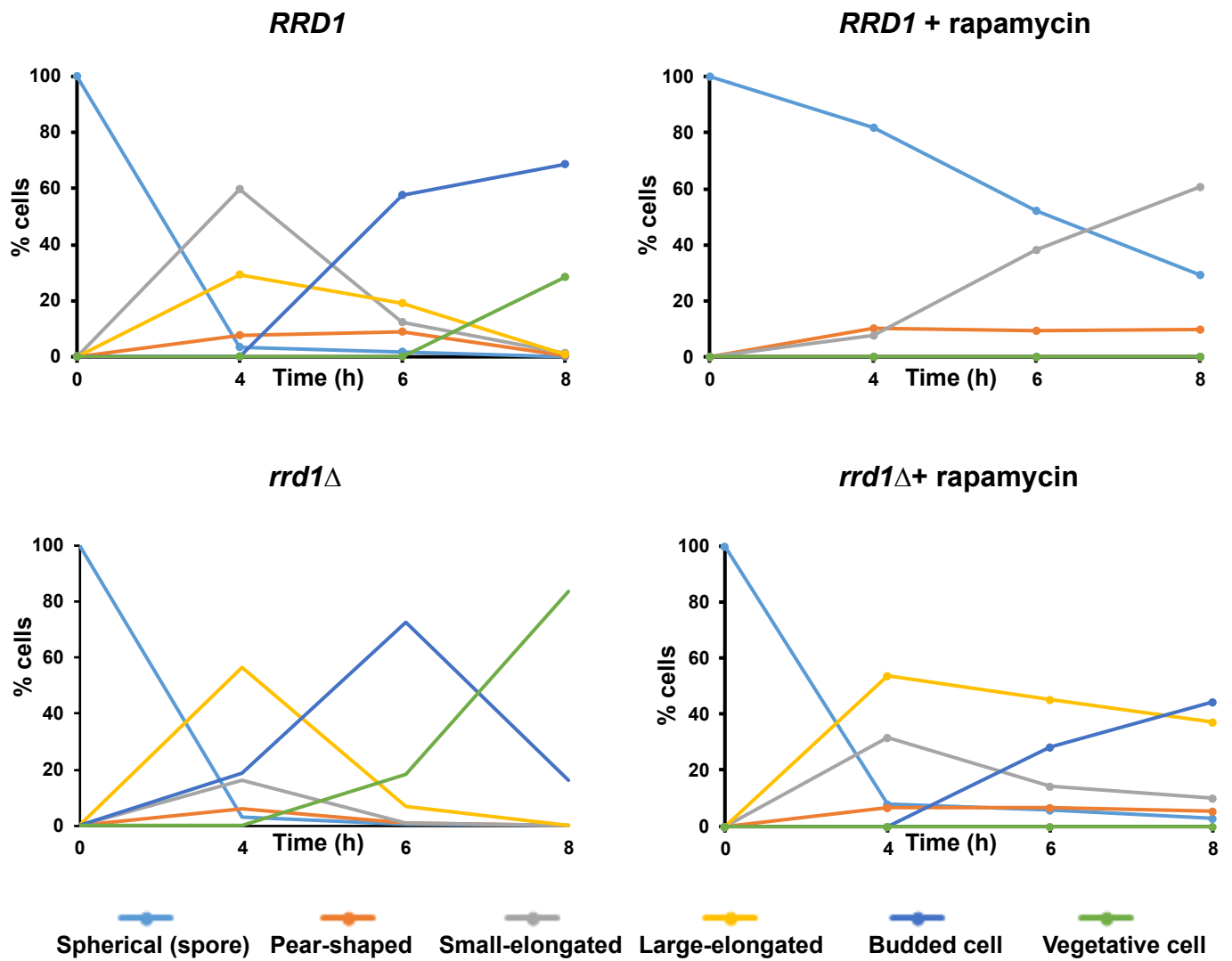

Figure S18

Supplement: Supplementary file 9 — Additional file 9: Fig. S14. Overlap of glucose-responsive genes with TORC1 target genes. a Comparison of our TORC1 target list identified during spore germination with those reported in a Micro-array-based study (Shamji et al.) and an RNA-Seq study (Gowans et al.) [ both performed with vegetative cells. b Comparison of the list of glucose-responsive genes (Wang et al.) with TORC1 target lists identified by our RNA-Seq analysis and an independent RNA-Seq study (Gowans et al.). Fig. S15. TORC1 regulates the glucose-responsive genes during spore germination. Spores were transferred to YPD medium with either DMSO or rapamycin (2 μM). Aliquots of yeast cells taken at the indicated time points (0 h, 0.5 h, 1 h, and 2 h) from the two cultures were used for preparing RNA. Expression of 7 TGC genes (GFD2, GPG1, UGA1, RME1, CIT1, CRC1 and DHR2) was assayed by Real-Time qRT-PCR analysis. Levels of transcripts were normalized with respect to actin mRNA. Data are presented as means ± standard deviation (n = 2 technical replicates). Fig. S16. TORC1 is required for spore germination. a Fourteen asci resulting from sporulation of wild type or fpr1Δ or TOR1-1 diploid cells were dissected on YPD + agar plates containing 1.5 μM rapamycin. Fourteen asci from wild type diploid cells were also dissected on a YPD agar plate without rapamycin. Percentage of budded cells was calculated by examining the spore morphology under the dissection microscope after 6 hours following dissection and is indicated in the plot. b Images of the agar plates described above following incubation at 30 °C for 2 days are presented. Fig. S17. TORC1 is required for expression of cell cycle genes during spore germination. Spores were transferred to YPD medium with either DMSO or rapamycin (2 μM). Aliquots of yeast cells taken at the indicated time points (0, 4, 6 and 7 h) from the two cultures were used for preparing RNA. Expression of cell cycle genes CLN1, CLN2, CLB6, EGT2, PCL1, PCL9, CHS2 and CTS1 was assaye [file 12915_2021_1030_MOESM9_ESM.pdf]
