# Supplementary material for: TORC1 regulates the transcriptional response to glucose and developmental cycle via the Tap42-Sit4-Rrd1/2 pathway in Saccharomyces cerevisiae
Source: BMC Biol. 2021 May 6;19:95. doi: 10.1186/s12915-021-01030-3 (PMC8103650; doi:10.1186/s12915-021-01030-3)
Supplement: Supplementary file 3 — Additional file 3: Fig. S4. TORC1 is required to maintain the expression of TGC genes in glucose-containing growth medium. Wild type cells grown into mid-log phase in YPD medium were treated with either DMSO or rapamycin (200 nM). Aliquots of the cultures were taken after 0, 0.5, 1 and 2 hours. RNA was extracted from the cultures and the expression of the TGC genes was analyzed by Real-Time qRT-PCR. Data are presented as means ± standard deviation (n = 2 replicates). Fig. S5. TORC1 regulates the glucose-responsive genes independently of Bcy1 T129 dephosphorylation. Cells expressing wild type Bcy1 and mutant bcy1- T129D were grown to logarithmic phase in SC-EG medium. Glucose was added to the cultures at the final concentration of 2% along with either rapamycin (200 nM) or DMSO. Aliquots of the cultures were taken after 0’, 15’ and 30’. RNA was extracted from the cultures and the expression of the glucose response genes DHR2, CIT1 and RME1 were analyzed by Real-Time qRT-PCR. Data are presented as means ± standard deviation (n = 2 replicates). Fig. S6. Inhibition of TORC1 has no detectable effect on overall PKA activity. Wild type (PKA) and pka-as cells were grown to logarithmic phase in YPD medium (2% glucose) and then either DMSO or rapamycin (200 nM) or 1-NM-PP1 (25 μM) was added to the cultures. a Whole cell extracts were prepared from aliquots of cells taken after 0, 1, 2 and 3 h and were analyzed by Western blotting using an anti-PKA substrate antibody and actin antibody (loading control). b RNA was extracted from aliquots of DMSO and rapamycin-treated cells taken after 0, 1, 2 and 3 h and the expression of DIP5 and GAP1 was analyzed by Real-Time qRT-PCR. Data are presented as means ± standard deviation (n = 2 technical replicates). Fig. S7. TORC1 regulates the expression of glucose-responsive genes independently of Sch9. Wild type and sch9Δ cells were grown to logarithmic phase in SC/EG medium and then glucose (2% final concentration) was added to the cultures [file 12915_2021_1030_MOESM3_ESM.pdf]

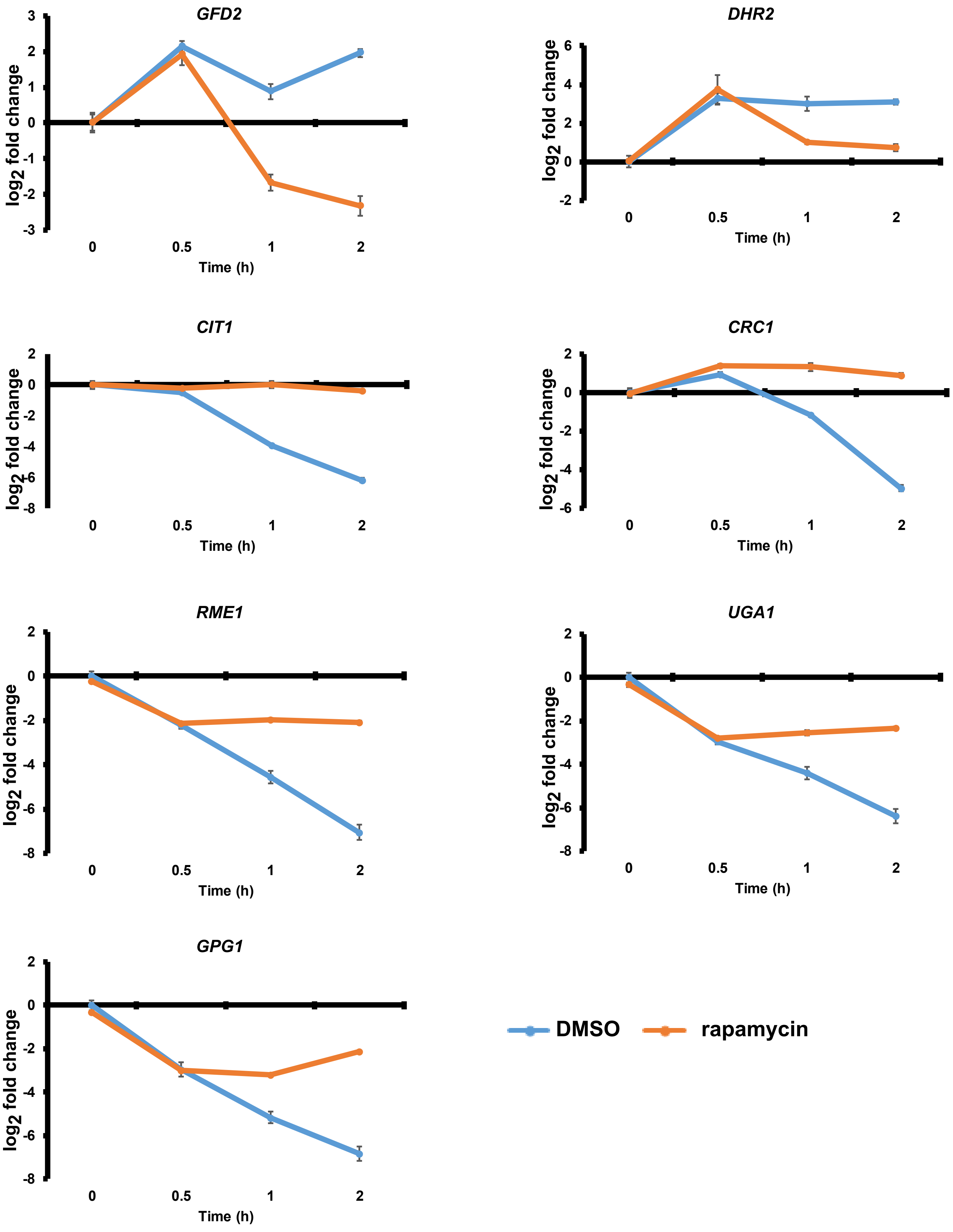
GFD2
DHR2
CIT1
CRC1
RME1
UGA1
GPG1
log2 fold change
Time (h)
0
0.5
1
2
DMSO
rapamycin

Figure S4

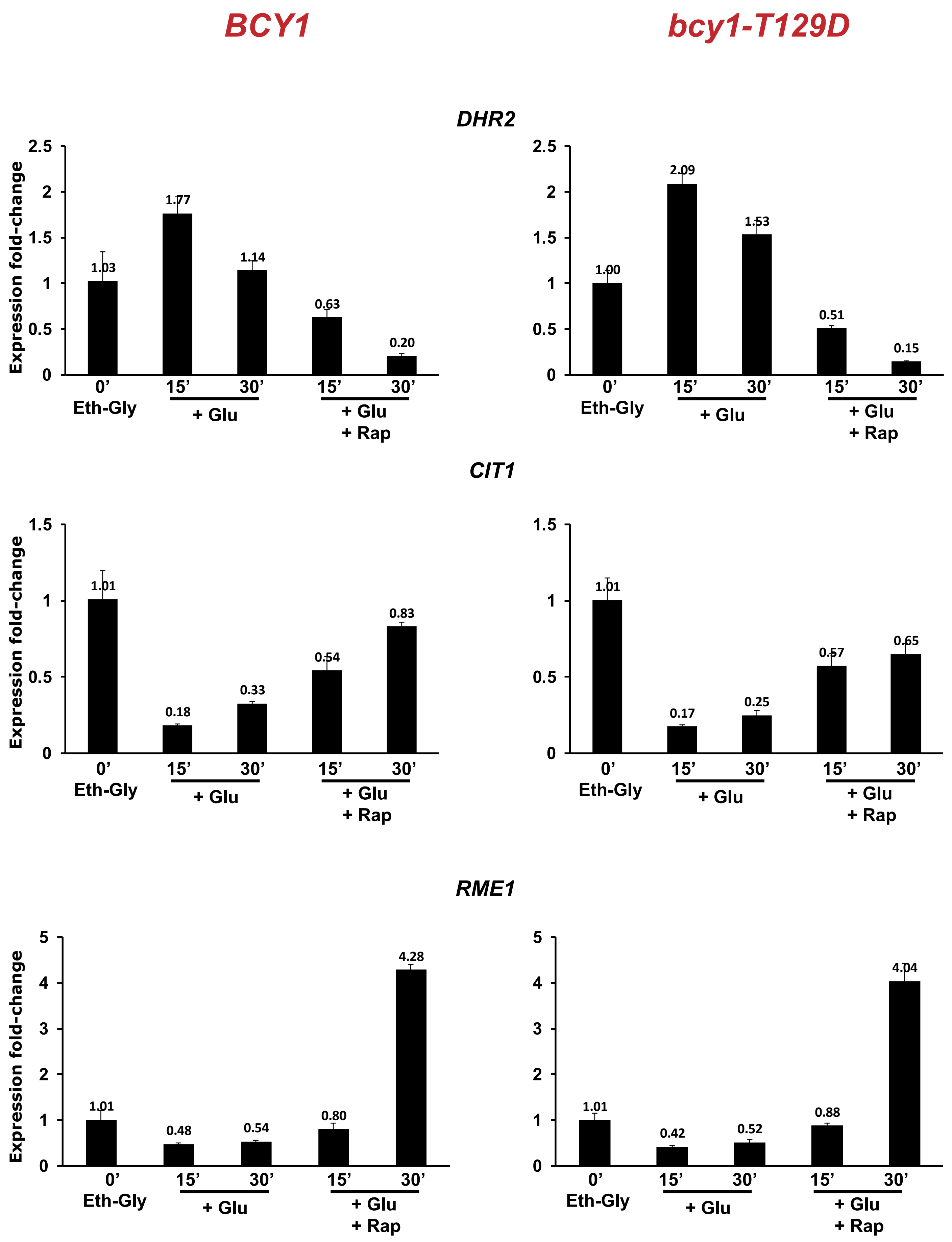
BCY1
bcy1-T129D
DHR2
CIT1
RME1
Expression fold-change
1.03
1.77
1.14
0.63
0.20
1.00
2.09
1.53
0.51
0.15
1.01
0.18
0.33
0.54
0.83
1.01
0.17
0.25
0.57
0.65
1.01
0.48
0.54
0.80
4.28
1.01
0.42
0.52
0.88
4.04
0’
Eth-Gly
15’
30’
+ Glu
+ Glu
+ Rap

Figure S5

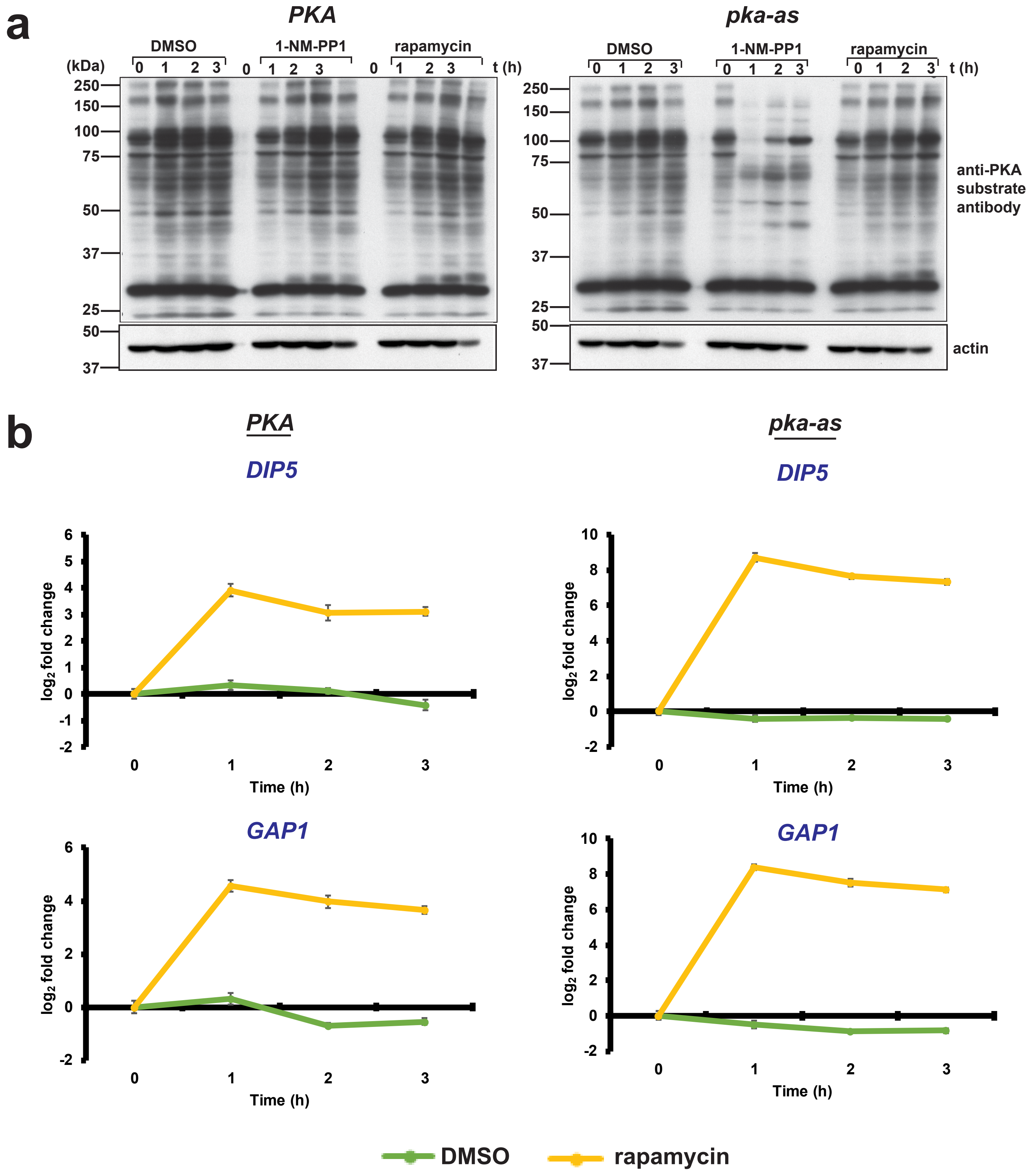
a
PKA
pka-as
DMSO
1-NM-PP1
rapamycin
(kDa)
t (h)
0 1 2 3
250
150
100
75
50
37
25
anti-PKA substrate antibody
actin
b
PKA
pka-as
DIP5
GAP1
log2 fold change
Time (h)
DMSO
rapamycin

Figure S6

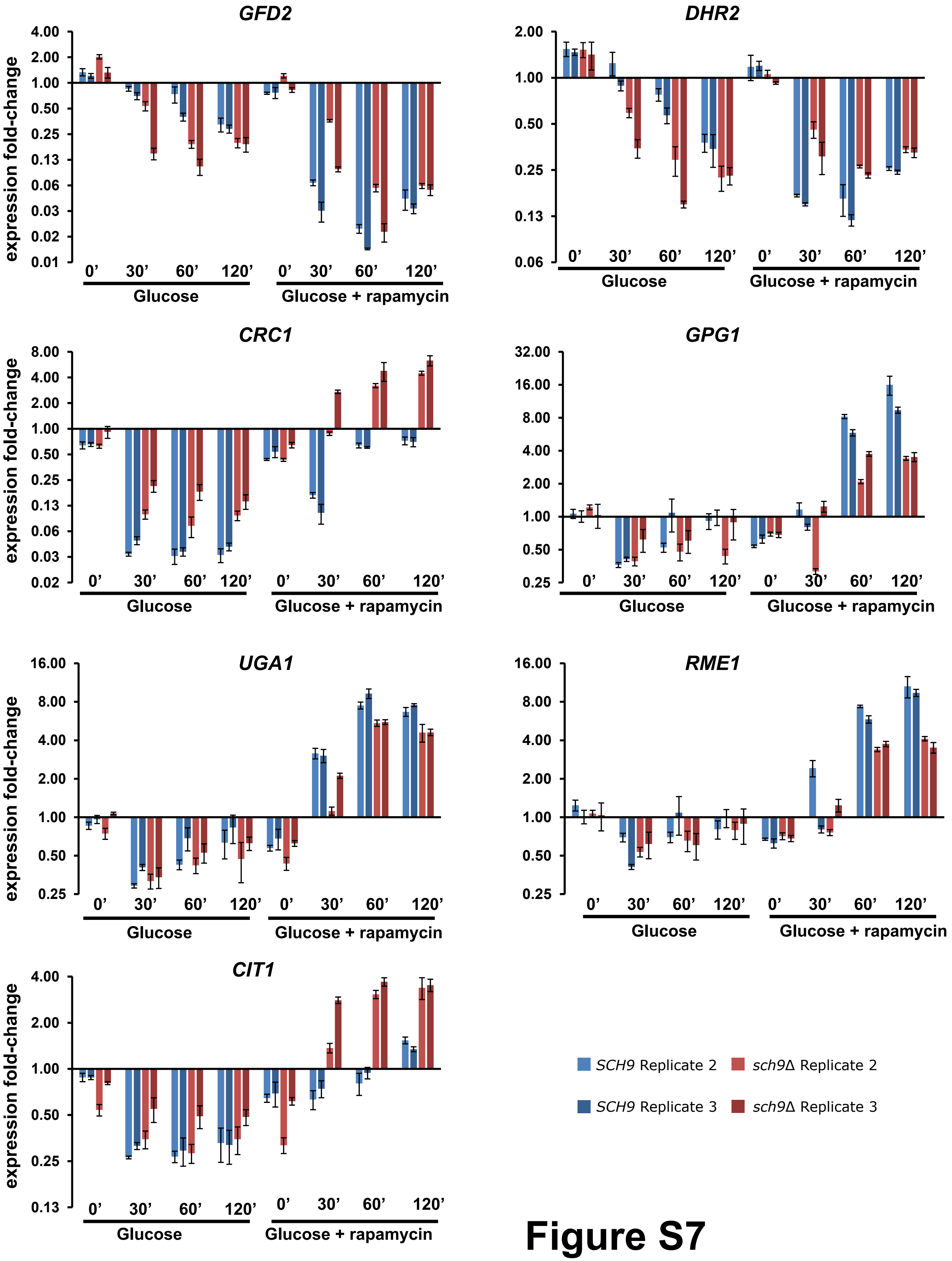
GFD2
DHR2
CRC1
GPG1
UGA1
RME1
CIT1
expression fold-change
4.00
2.00
1.00
0.50
0.25
0.13
0.06
0.03
0.02
0.01
8.00
16.00
32.00
0'
30'
60'
120'
Glucose
Glucose + rapamycin
SCH9 Replicate 2
SCH9 Replicate 3
sch9Δ Replicate 2
sch9Δ Replicate 3

Figure S7

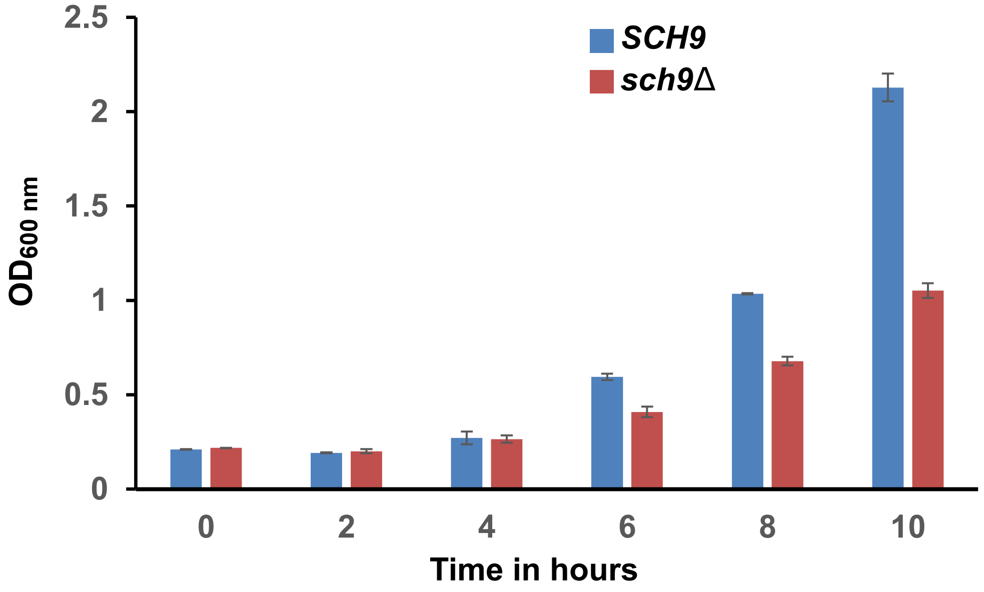

Figure S8

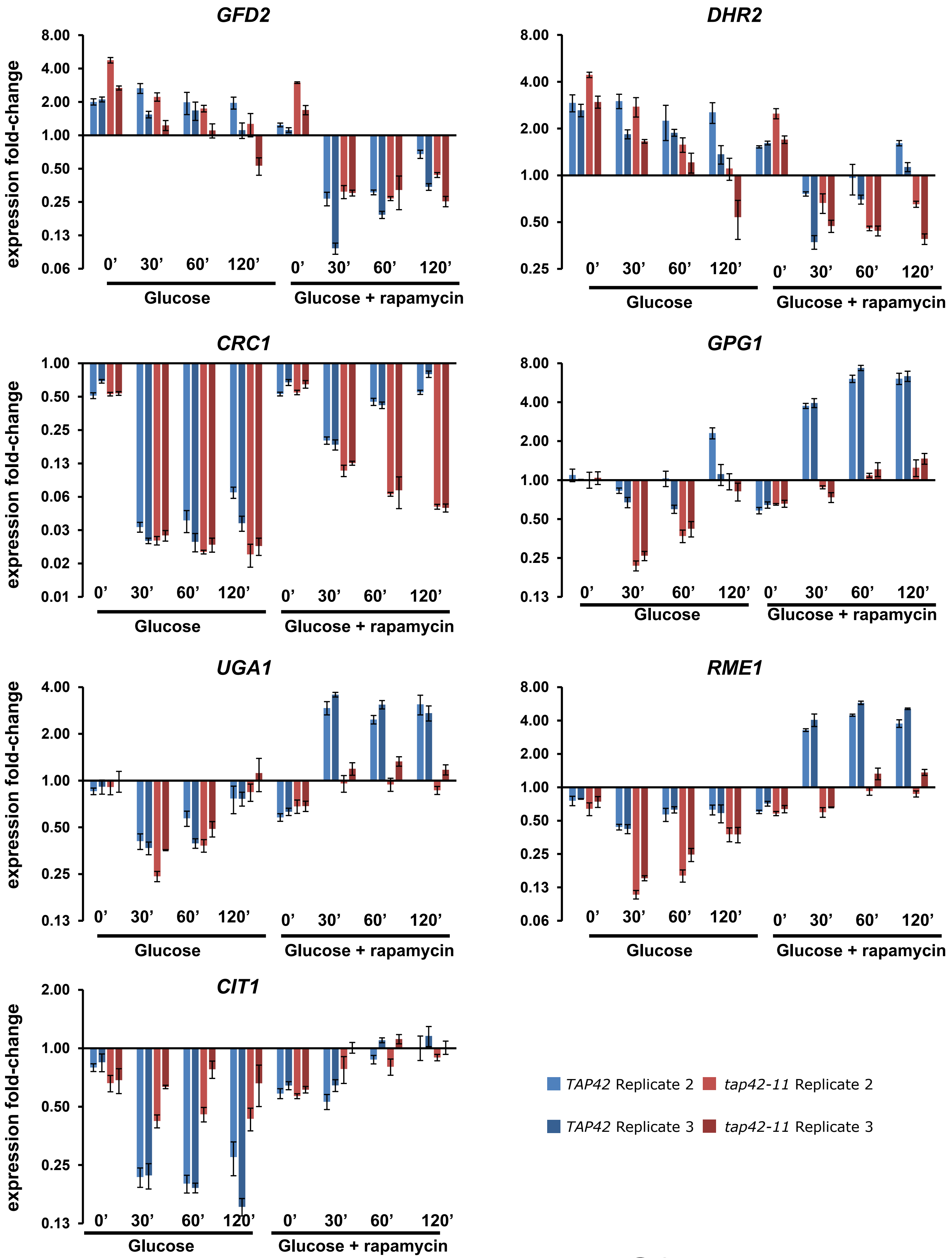
GFD2
DHR2
CRC1
GPG1
UGA1
RME1
CIT1
expression fold-change
8.00
4.00
2.00
1.00
0.50
0.25
0.13
0.06
0.03
0.02
0.01
0'
30'
60'
120'
Glucose
Glucose + rapamycin
TAP42 Replicate 2
tap42-11 Replicate 2
TAP42 Replicate 3
tap42-11 Replicate 3

Figure S9

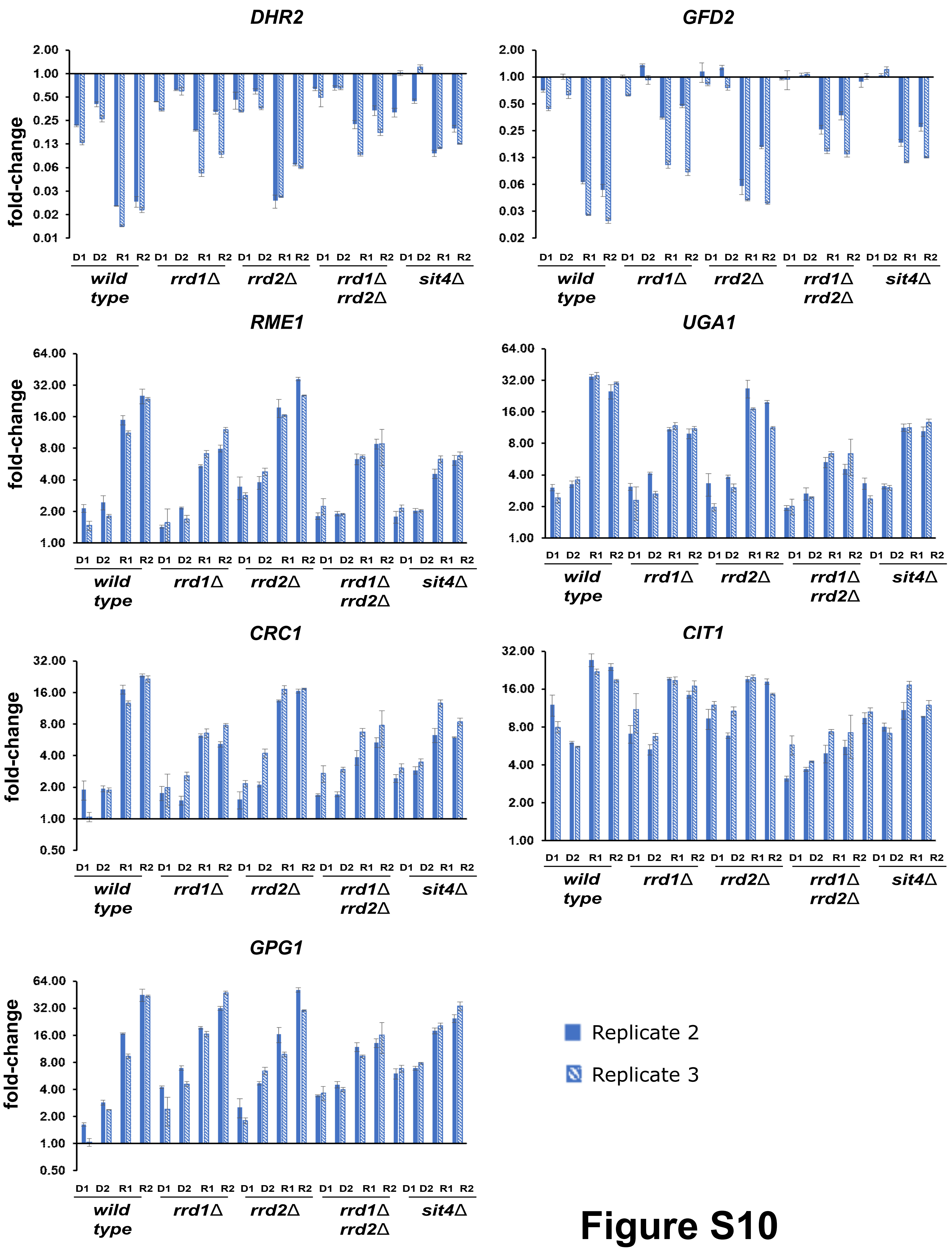
DHR2
fold-change
2.00
1.00
0.50
0.25
0.13
0.06
0.03
0.02
0.01
GFD2
2.00
1.00
0.50
0.25
0.13
0.06
0.03
0.02
RME1
fold-change
64.00
32.00
16.00
8.00
4.00
2.00
1.00
UGA1
64.00
32.00
16.00
8.00
4.00
2.00
1.00
CRC1
fold-change
32.00
16.00
8.00
4.00
2.00
1.00
0.50
CIT1
32.00
16.00
8.00
4.00
2.00
1.00
GPG1
fold-change
64.00
32.00
16.00
8.00
4.00
2.00
1.00
0.50
D1 D2 R1 R2 D1 D2 R1 R2 D1 D2 R1 R2 D1 D2 R1 R2 D1 D2 R1 R2
wild type
rrd1Δ
rrd2Δ
rrd1Δ rrd2Δ
sit4Δ
Replicate 2
Replicate 3

Figure S10

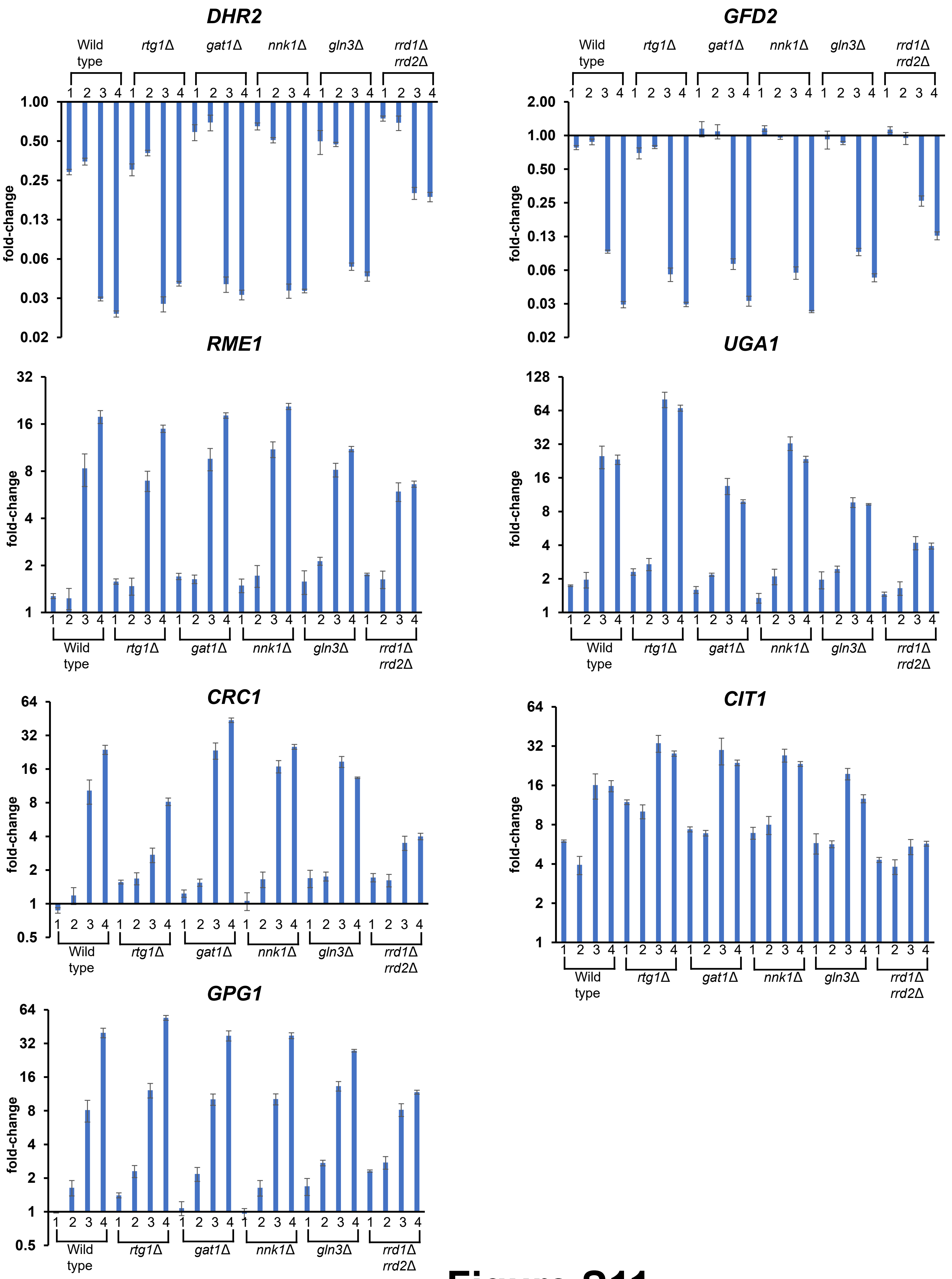
DHR2
GFD2
RME1
UGA1
CRC1
CIT1
GPG1
fold-change
Wild type
rtg1Δ
gat1Δ
nnk1Δ
gln3Δ
rrd1Δ rrd2Δ
1 2 3 4
1.00
0.50
0.25
0.13
0.06
0.03
0.02
2.00
128
64
32
16
8
4
2
1
0.5

Figure S11

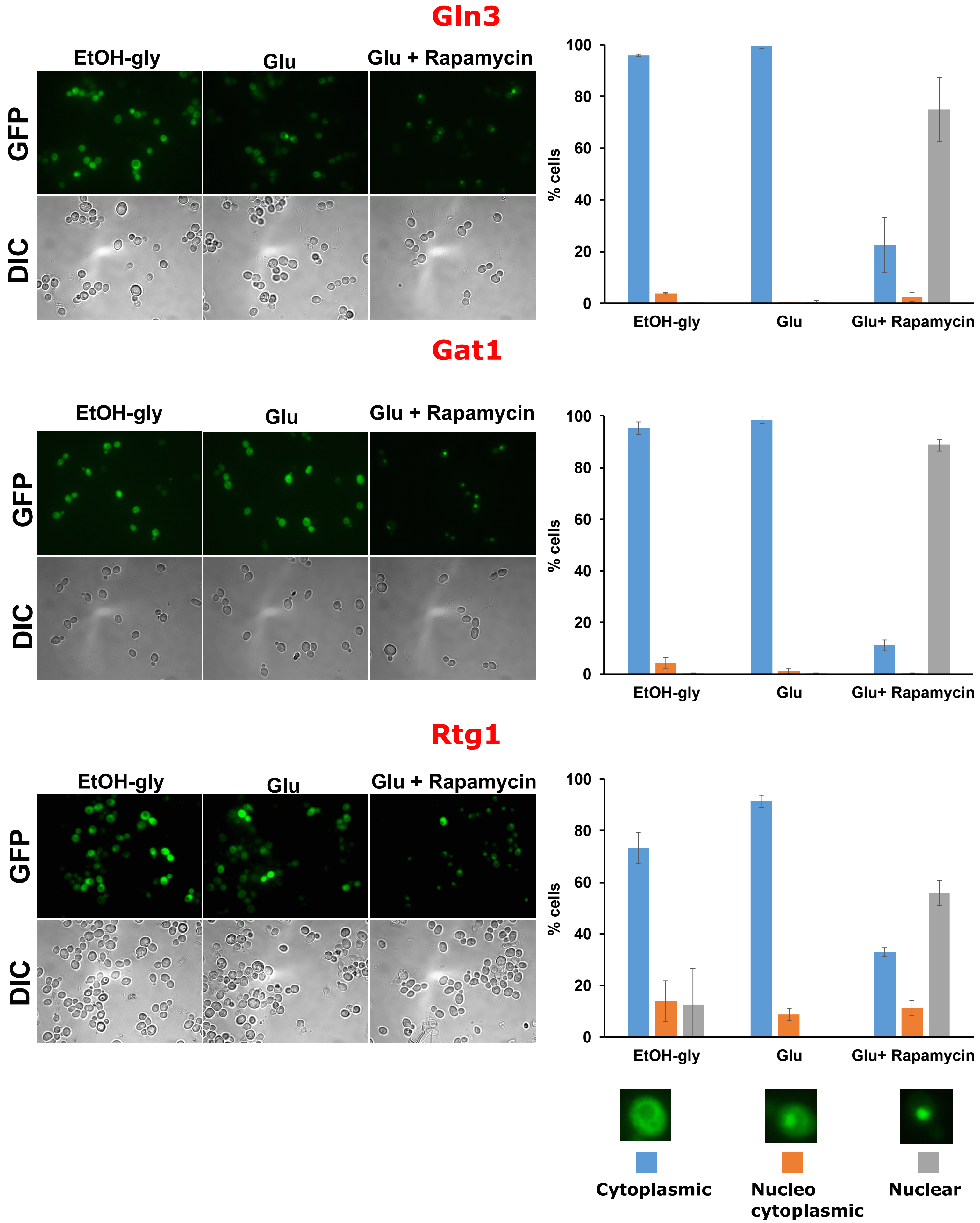

Gln3
EtOH-gly
Glu
Glu + Rapamycin
GFP
DIC
% cells
100
80
60
40
20
0
EtOH-gly
Glu
Glu+ Rapamycin
Gat1
Rtg1
Cytoplasmic
Nucleo cytoplasmic
Nuclear

Figure S12
