# Supplementary material for: TORC1 regulates the transcriptional response to glucose and developmental cycle via the Tap42-Sit4-Rrd1/2 pathway in Saccharomyces cerevisiae
Source: BMC Biol. 2021 May 6;19:95. doi: 10.1186/s12915-021-01030-3 (PMC8103650; doi:10.1186/s12915-021-01030-3)
Supplement: Supplementary file 10 — Additional file 10: Table S6. A list of yeast strains used in the study. Table S7. A list of primers used for Real-Time qRT-PCR analyses in the study. [file 12915_2021_1030_MOESM10_ESM.docx]

**Table S6 List of strains used in the study**

All strains are derivatives of SK1 and have the following markers ho::LYS2 ura3 leu2 trp1 his3 lys2 All markers are homozygous in diploid strains unless otherwise mentioned

| Strain number | Genotype | Used in Figure |
| --- | --- | --- |
| 3526 | MATa *sch9: SCH9-HA6::KANMX6* | 2,3,4, 5, S1, S2, S3, S4, S6 |
| 3681 | MATa *gtr1:KANMX6* *sch9: SCH9-HA6::KANMX6* | 2, 3 |
| 3542 | MATa *sch9: SCH9-HA6::KANMX6 fpr1:NatMX6* | 2 |
| 4670 | MATa *gtr1:KANMX6* *sch9: SCH9-HA6::KANMX6 fpr1:NATMX6* | 2 |
| 3576 | MATa *sch9: SCH9-HA6::KANMX6 tpk1-as tpk2-as tpk3:KanMX6* | 2, 4, S1, S2, S3, S6 |
| 3772 | MATa *sch9: SCH9-HA6::KANMX6 rgt2::HIS3MX6* | 2 |
| 3780 | MATa *sch9: SCH9-HA6::KANMX6 snf3::HIS3MX6* | 2 |
| 3784 | MATa *sch9: SCH9-HA6::KANMX6 rgt2::HIS3MX6 snf3:HIS3MX6* | 2 |
| 1150 | MATa | 5, 7, S7, S8, S10, S11 |
| 3502 | MATa *sch9:KANMX6* | 5, S7, S8 |
| 3569 | MATa *tap42:KANMX6 TAP42-CEN- LEU2* | 6, S9 |
| 3571 | MATa *tap42:KANMX6 tap42-11-CEN- LEU2* | 6, S9 |
| 4860 | MATa *rrd1:NATMX6* | 7, S10, |
| 3530 | MATa *rrd2:KANMX6* | 7, S10, |
| 4861 | MATa *rrd1:NATMX6 rrd2:NATMX6* | 7, S10, S11 |
| 3249 | MATa *sit4:HIS3MX6* | 7, S10, |
| 3555 | MATa/MATα *sch9: SCH9-HA6::KANMX6* | 8 |
| 1738 | MAT a/MATα | 8, 9, S13, S15, S16, S17, S18 |
| 3478 | MAT a/MATα *TOR1-1* | 9, S16 |
| 4344 | MATa *bcy1:KANMX6 YCplac33::HA-BCY1 (CEN URA3)* | S5 |
| 4347 | MATa *bcy1:KANMX6 YCplac33::HA- bcy1--T129D (CEN URA3)* | S5 |
| 4349 | MATa *bcy1: KANMX6YCplac33::HA-- bcy1--S145A (CEN URA3)* | S5 |
| 5088 | MATa *rtg1:NATMX6* | S11 |
| 5092 | MATa *gat1:NATMX6* | S11 |
| 5090 | MATa *nnk1:NATMX6* | S11 |
| 5087 | MATa *gln3:KANMX6* | S11 |
| 3477 | MATa /MATα *sch9: SCH9-HA6::KANMX6 fpr1:NATMX6* | S13 |
| 4885 | MATa /MATα *rrd1:NATMX6* | S18 |
| 5144 | MATa pRS316-GLN3-GFP | S12 |
| 5145 | MATa pRS316-GAT1-GFP | S12 |
| 5146 | MATa pRS316-RTG1-GFP | S12 |

**Table S7. List of primers used for RT-qPCR**

| **Primer** | **Gene (S=forward & A=reverse)** | **Sequence (5' -> 3')** |
| --- | --- | --- |
| MA71 | ACT1_S | GAAGTGTGATGTCGATGTCC |
| MA72 | ACT1_A | TCTTTCTGGAGGAGCAATG |
| MA73 | GAP1_S | ATCCTTCCCACTTGTTATGG |
| MA74 | GAP1_A | GCCTTTTCTTCTGCAATTTC |
| MA75 | DIP5_S | TCAAAATTTGCTTATGTCGC |
| MA76 | DIP5_A | AGACAGGCAGGCCAATATAC |
| MA77 | TPS2_S | ATCAATGGGGCAACTACG |
| MA78 | TPS2_A | CGTACCAGCACTTTGGAAG |
| MA79 | GCD10_S | GACCTCAGGTTTTTAGCACC |
| MA80 | GCD10_A | TCCGATACAGGTTCAGGAG |
| MA81 | SPB4_S | ACTCGAGAGAAAGGAAAAGATG |
| MA82 | SPB4_A | GATAGCTTTGCTGGAAACTTTC |
| MA83 | YOL014W_S | TCTACTTGGCATGGTGTCC |
| MA84 | YOL014W_A | CATATTCGTTGGCTTCAGTG |
| MA85 | GFD2_S | ACCCTGCATTTGTTCATG |
| MA86 | GFD2_A | TCGATGGAGATGGCATAC |
| MA87 | DHR2_S | CTATAGGGATGCCAGACAGG |
| MA88 | DHR2_A | CATTTCTGGCATATCCCTTC |
| MA89 | GDH1_S | TTCTGGTTTAGAAATGGCAC |
| MA90 | GDH1_A | TTGACCAAAGATGGCAAG |
| MA91 | CLB6_S | GCATTGAAACAAGGAACATG |
| MA92 | CLB6_A | AAAAGTTTCATCCCATTTGG |
| MA93 | TOS6_S | ATGTTACCACCACCCCAC |
| MA94 | TOS6_A | CCGACGTATGTGCTGATC |
| MA95 | PRM7_S | CGTTCAATCAACTGCTTCC |
| MA96 | PRM7_A | CGTTAGTAGTGGTGGTCGTG |
| MA97 | ZRT1_S | GTGTTTTGGATGCCATTTC |
| MA98 | ZRT1_A | AAAGCCATGATACCAGCAC |
| MA99 | CRC1_S | TTGAACGTGTGTCTTGCTG |
| MA100 | CRC1_A | CCCTTGATACCACCTCTTTG |
| MA101 | CIT1_S | GTTGGTCTCCACCATTTATG |
| MA102 | CIT1_A | TGGCAACACCAAACAATAC |
| MA103 | RME1_S | GTCCCATAGAGCAATGTCC |
| MA104 | RME1_A | AAATGGGCAATTCAGTCC |
| MA105 | UGA1_S | GGGTTTGCAGAAGAAATACC |
| MA106 | UGA1_A | GACTGCACATCCACCAAC |
| MA107 | PCL9_S | AATACCAACCGTCCCTACC |
| MA108 | PCL9_A | ACTGGTGAACTTCCCATTG |
| MA109 | GPG1_S | TCGGAGAGTTGCACACAC |
| MA110 | GPG1_A | TTCATTGAAAGCACATCCC |
| MA111 | CHS2_S | AATTGTGATGATTTGGATGC |
| MA112 | CHS2_A | ACAAAAAGGCCATGGAAC |
| MA113 | PCL1_S | TGGCACGAGTCCTATCAG |
| MA114 | PCL1_A | CTGTGTTGTTCGCTATGTTG |
| MA115 | CTS1_S | ATATGCGGCTGGTAAATTG |
| MA116 | CTS1_A | AGTCGCCGGAGTCATAAG |
| MA117 | EGT2_S | GCCGAGCACACAAATTTAG |
| MA118 | EGT2_A | TGGAACTTGTTGGCAATG |
| MA119 | CLN1_S | TGCCATAAGCGTAAGCAG |
| MA120 | CLN1_A | GTCATAAATTTGGCACGTTG |
| MA121 | CLN2_S | AGCAATAACGCAACCAATG |
| MA122 | CLN2_A | TTTATGGTCCCAGTTGGC |
| MA123 | DSE1_S | ACCGGTAAAATAATCGATGG |
| MA124 | DSE1_A | ATTTGCTGAAGAACAATCCC |
| MA125 | DSE2_S | CGTCATCGTCTTCCACTTC |
| MA126 | DSE2_A | AGACGGATATCGATTGCG |
| MA127 | DSE3_S | AGTTGCACAAACATCATCG |
| MA128 | DSE3_A | CAAAGCCCTATCCTCGTC |
| MA129 | DSE4_S | ATTGGTTCAACGGTCATTC |
| MA130 | DSE4_A | TCAAGTCACCCCTCAATTC |
| MA131 | SCW11_S | GAAACTGCCGGTACATTTG |
| MA132 | SCW11_A | TTTGGACTGCAGTAATTTGG |
| MA133 | HXT1_S | CTACGGTTACGTTTTCATGG |
| MA134 | HXT1_A | CTTGGATACTGGAACCCAG |
| MA135 | FBP1_S | CCTTTTCGCATACCCTTG |
| MA136 | FBP1_A | TCATGGATATGACTTGGCAC |
| MA137 | AQR1_S | GGTGCAGTTTGAGTACCATC |
| MA138 | AQR1_A | ATCCTCCCTCCACTTCATAC |
| MA139 | JEN1_S | CATCTTCACATTTGCTTGTG |
| MA140 | JEN1_A | CATTCCGTCTTTTGTTCAAC |
| MA141 | YIG1_S | CATGCTTCAGGAACAGGAC |
| MA142 | YIG1_A | CCGTATTTGGCCCTAATG |
| MA143 | MIG2_S | CACCACCAAGACTAGGAGG |
| MA144 | MIG2_A | TACGCCTGACAATTTCTTTC |
